# Cooling photon-pressure circuits into the quantum regime

**DOI:** 10.1126/sciadv.abg6653

**Published:** 2021-10-15

**Authors:** Ines Corveira Rodrigues, Daniel Bothner, Gary Alexander Steele

**Affiliations:** 1 Kavli Institute of Nanoscience, Delft University of Technology, PO Box 5046, 2600 GA Delft, Netherlands.; 2 Physikalisches Institut, Center for Quantum Science (CQ) and LISA^+^, Universität Tübingen, 72076 Tübingen, Germany.

## Abstract

Quantum control of electromagnetic fields was initially established in the optical domain and has been advanced to lower frequencies in the gigahertz range during the past decades extending quantum photonics to broader frequency regimes. In standard cryogenic systems, however, thermal decoherence prevents access to the quantum regime for photon frequencies below the gigahertz domain. Here, we engineer two superconducting *LC* circuits coupled by a photon-pressure interaction and demonstrate sideband cooling of a hot radio frequency (RF) circuit using a microwave cavity. Because of a substantially increased coupling strength, we obtain a large single-photon quantum cooperativity 𝒞_q0_ ∼ 1 and reduce the thermal RF occupancy by 75% with less than one pump photon. For larger pump powers, the coupling rate exceeds the RF thermal decoherence rate by a factor of 3, and the RF circuit is cooled into the quantum ground state. Our results lay the foundation for RF quantum photonics.

## Teaser

We demonstrate sideband cooling to the ground state and quantum coherent photon-pressure coupling with a hot electronic circuit.

## INTRODUCTION

In the recent decade, the parametric radiation-pressure coupling between two harmonic oscillators has been demonstrated to allow for groundbreaking experiments in the control and detection of harmonic oscillators from the kilohertz to the gigahertz frequency regime (*1*–*8*). The archetype of a radiation-pressure–coupled system is an optomechanical cavity (*1*), where the interaction between a mechanical oscillator and light inside an electromagnetic resonator is used for displacement sensing and motion control of macroscopic objects with unprecedented precision. An outstanding feature of the radiation-pressure interaction is the possibility to cool low-frequency a mechanical oscillator orders of magnitude below its thermodynamic bath temperature using cavity red-sideband driving (*9*–*15*). The application of this technique to trapped ions and atoms (*16*, *17*) or to mechanical oscillators has been used to place them in the phononic ground state (*18*–*23*) and is the prerequisite for the preparation and investigation of quantum states of motion (*3*, *5*, *24*).

The implementation of photon-pressure coupling—radiation-pressure coupling between two photons of different frequency—using superconducting *LC* circuits has recently gained considerable attention (*25*–*29*), as its realization promises to enable quantum control of electromagnetic fields in the radio frequency (RF) domain. This platform might also allow for the investigation of unexplored photon-pressure parameter regimes, as circuits provide an extremely high degree of design flexibility regarding resonance frequencies and linewidths. Radiation-pressure–coupled devices provide furthermore extensive possibilities for quantum signal processing, such as quantum-limited parametric amplification (*30*–*34*), nonreciprocal photon transport (*8*, *35*–*37*), slow light (*38*, *39*), and photonic reservoir engineering (*40*, *41*). Implementing these technologies in a circuit-only platform would not only offer larger coupling rates and more architectural possibilities compared to mechanical oscillators but would also be naturally compatible with superconducting quantum processors (*42*). Very recently, photon-pressure–coupled circuits are also discussed as in the context of fault-tolerant quantum computing using bosonic codes (*29*) and quantum-enhanced dark matter axion detection at low-energy scales (*43*, *44*). To date, however, photon-pressure–coupled superconducting circuits have only been realized in the classical regime and in the presence of large residual thermal fluctuations (*25*, *26*).

Here, we report photon-pressure coupling between a hot RF circuit and a high-frequency (HF) superconducting quantum interference cavity in the quantum regime. By engineering galvanically connected circuits, we increase the single-photon coupling strength and single-photon cooperativity by about one order of magnitude compared to the best results reported to date (*25*, *26*). This allows for sideband cooling of the residual thermal occupation in the hot RF mode by a factor of ∼4 with less than one pump photon and a single-photon quantum cooperativity 𝒞_q0_ ∼ 1. Because of the large single-photon coupling rate in our device, we reach the strong-coupling regime with only 0.7 pump photons, where we observe the residual thermal fluctuations of the hybridized normal modes and demonstrate ground state cooling of the RF mode. Simultaneuosly, the multiphoton coupling rate notably exceeds the thermal decoherence rate of the RF mode and the decay rate of the HF cavity, which corresponds to the quantum-coherent strong-coupling regime, the basis for coherent quantum-state transfer between the two circuits (*45*). Our results pave the way toward quantum control of RF circuits and quantum-limited detection of photons in the RF regime.

## RESULTS

**Fig. 1.**
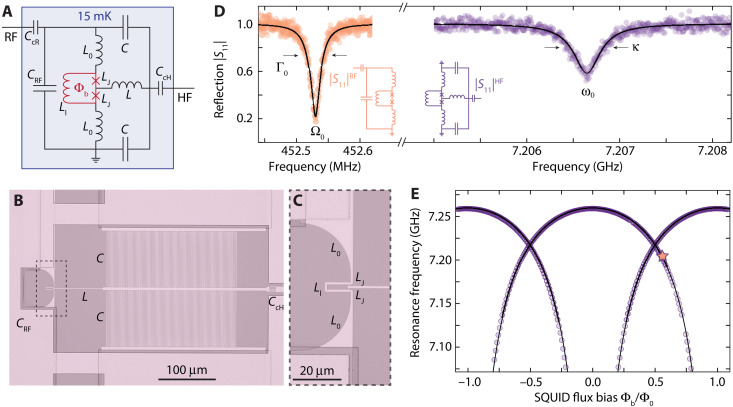
A two-mode superconducting *LC* circuit with a tunable photon-pressure interaction. (**A**) Circuit schematic. The full circuit has two modes, a low-frequency mode and an HF mode. The low, RF mode is formed by the capacitors and inductors *C*_RF_ and *L*_0_ and the parallel combination of *L*_l_ and 2*L*_J_. The HF microwave mode is formed by the combination of *L*, *C*, and *L*_0_ and *L*_J_. The inductances *L*_l_ and *L*_J_ form a SQUID. Both modes are capacitively coupled to individual feedlines for driving and readout. (**B**) Optical image of the device showing the circuit (full image is shown in fig. S2). The dashed box shows the zoom region for (**C**). In (B) and (C), brighter parts correspond to aluminum, and darker and transparent parts correspond to silicon. (**D**) Resonance curves of both modes versus excitation frequency in the |S_11_|; colored points are data, and the black lines correspond to fits. The inset displays the reduced circuit equivalents for the two modes. (**E**) Resonance frequency of the HF mode versus magnetic flux bias through the SQUID loop generated by an external magnetic coil. The dataset was obtained by combining data from a flux up-sweep with the data from a flux down-sweep. Because of a non-negligible loop inductance *L*_l_, the flux dependence is hysteretic and multivalued for flux values around ±0.5Φ_0_ ± 0.3Φ_0_ (*60*–*62*). The flux operation point Φ_b_ = Φ_0_ ~ 0.54 used for the data shown in (D) and for the rest of this work is marked by a star.

Our device combines two integrated superconducting *LC* circuits, which are galvanically connected to each other at the heart of the circuit in a superconducting quantum interference device (SQUID). A circuit schematic of the device and optical micrographs are shown in Fig. 1 (A to C), and the multilayer device fabrication is presented in detail in note S1.

The RF mode circuit consists of a large parallel-plate capacitor using amorphous silicon as a dielectric and of a short inductor wire, which at the same time forms the loop of the SQUID. The SQUID is completed by two constriction type Josephson junctions connecting the RF inductor wire to the HF part of the circuit. The remaining part of the HF mode consists of an additional linear inductor *L* and two interdigitated capacitors *C*; cf. Fig. 1. Both circuit modes are capacitively coupled to individual coplanar waveguide feedlines for driving and readout. The chip is mounted into a printed circuit board, connected to microwave input/output cabling, and packaged into a radiation tight copper (Cu) housing. A small superconducting magnet is attached to the Cu housing below the chip allowing the application of an external out-of-plane magnetic field. The experiments are carried out with the whole configuration placed inside a cryoperm magnetic shielding and attached to the mixing chamber of a dilution refrigerator with a base temperature of *T*_b_ ∼ 15 mK. More details on the device and the setup are given in notes S2 and S3 (*46*–*48*).

In Fig. 1D, the reflection response ∣*S*_11_∣ of the two modes is shown, measured through their individual feedlines. The RF mode has a resonance frequency of Ω_0_ = 2π · 452.5 MHz and a linewidth Γ_0_ = 2π · 26 kHz. For the HF mode, the resonance frequency is ω_0_ = 2π · 7.207 GHz and the total linewidth κ = 2π · 380 kHz. The total linewidth of the HF mode is the sum of the internal contribution κ_i_ = 2π · 300 kHz and the external contribution due to the coupling to the feedline of κ_e_ = 2π · 80 kHz. For the low-frequency circuit, we obtain Γ_i_ = 2π · 10 kHz and Γ_e_ = 2π · 16 kHz. Details on the fitting function and routine are given in note S4.

When a magnetic flux Φ_b_ is applied through the SQUID by the external coil, the resulting circulating current changes the inductance of the Josephson junctions and the HF resonance frequency is shifted accordingly. In Fig. 1E, we show ω_0_(Φ_b_) depending on the external bias flux Φ_b_ through the SQUID loop; a theoretical description and modeling of the circuit and the flux dependence are detailed in note S3. Any oscillating current flowing through the RF inductor induces additional flux through the SQUID loop and therefore modulates the resonance frequency of the HF mode. As a result, the two modes interact via an effective photon-pressure coupling and the Hamiltonian of the device is given by (*25*–*27*)

$$\hat{H}/\hbar =\omega_{0}{\hat{a}}^{†}\hat{a}+\Omega_{0}{\hat{b}}^{†}\hat{b}+g_{0}{\hat{a}}^{†}\hat{a}(\hat{b}+{\hat{b}}^{†})$$ (1)

where the creation (annihilation) operators for the HF and RF modes are given by ${\hat{a}}^{†}$ ($\hat{a}$) and ${\hat{b}}^{†}$ ($\hat{b}$), respectively. The coupling constant is given by

$$g_{0}=\frac{\partial \omega_{0}}{\partial \Phi_{b}}\Phi_{\text{zpf}}$$ (2)

with the tunable flux responsivity of the HF cavity ∂ω_0_/∂Φ_b_ and the effective root mean square value of the RF vacuum flux fluctuations Φ_zpf_ ≈ 635 μΦ_0_. Because of the low-power operation regime used for the experiments presented here, both circuits act in good approximation as harmonic oscillators, and the Kerr nonlinearities arising from the Josephson junctions can be neglected. With the small Josephson inductance *L*_J0_ = 40 pH of the constriction type junctions and because of the dilution by linear inductors, both Kerr nonlinearities χ_HF_ = 2π · 2.4 kHz ≪ κ and χ_RF_ = 2π · 1.3 Hz ≪ Γ_0_ (*49*) are sufficiently low to justify this approximation.

From the resonance frequency fit curves shown in Fig. 1E, the flux responsivity at the operation point is found to be ∂ω_0_/∂Φ ≈ 2π · 250 MHz/Φ_0_, and we get a coupling rate of *g*_0_ = 2π* ·* 160 kHz at the operation point. At larger flux bias values Φ_b_/Φ_0_ ∼ 0.75, the single-photon coupling rate reaches values *g*_0_ ∼ 2π · 1 MHz ≈ κ; cf. note S5, a regime typically very difficult to reach in other photon-pressure–coupled systems. In the current setup, however, this operation point is related to considerable low-frequency flux noise, which leads to HF cavity fluctuations and slow frequency drifts. Therefore, we chose to work at an operation point that simultaneously offers a large single-photon coupling rate and negligible HF cavity fluctuations.

Considering the parameter regime of our device with *g*_0_/Ω_0_ ∼ 3 · 10^−4^, the photon-pressure nonlinearity (*50*, *51*) induced in the HF cavity given by $2g_{0}^{2}/\Omega_{0}∼2\pi \cdot 110$Hz is negligibly small. Therefore, the interaction between the two modes with a coherently driven HF cavity can be linearized (*1*), and the interaction part of the Hamiltonian with red-sideband driving is captured by a pump-tunable beam-splitter interaction

$${\hat{H}}_{\text{int}}/\hbar =g(\delta \hat{a}{\hat{b}}^{†}+\delta {\hat{a}}^{†}\hat{b})$$ (3)

Here, $g=\sqrt{n_{c}}g_{0}$ is the multiphoton coupling strength and $\delta {\hat{a}}^{†}$ and $\delta \hat{a}$ describe the creation and annihilation of intracavity field fluctuations, respectively. In this situation, photons from the pump will scatter mainly to the HF resonance frequency ω_0_, each event removing one photon from the RF circuit. This process constitutes a cooling mechanism, which is exhibited by an additional damping term of the RF mode.

**Fig. 2.**
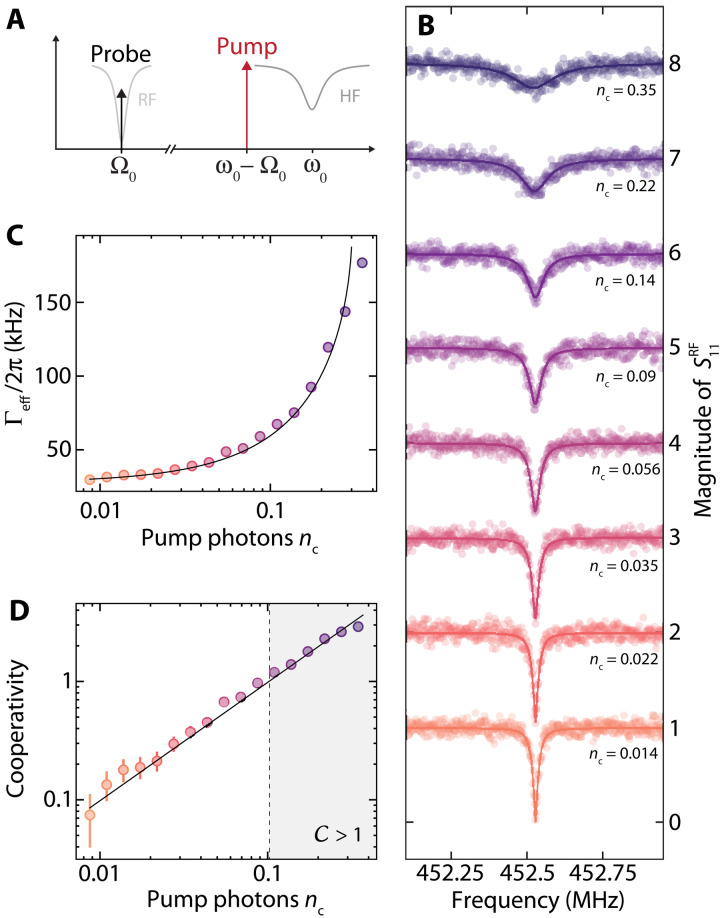
Photon-pressure damping of the RF mode and large single-photon cooperativity. (**A**) Schematic of the experiment. The HF mode is driven by a pump tone on its red sideband ω_p_ = ω_0_ − Ω_0_, and the response of the RF mode is simultaneously measured with a weak probe tone around Ω ∼ Ω_0_. With increasing strength of the pump tone or intracavity pump photon number *n*_c_, respectively, the linewidth of the RF resonance broadens substantially as shown in (**B**), indicating the regime of photon-pressure damping induced by the red-sideband pump field. Circles are data, and lines are fits. Subsequent curves are shifted vertically by 1 for clarity. From the fits, we extract the effective RF mode linewidth Γ_eff_ depending on the number of intracavity pump photons. The extracted values are plotted in (**C**). By fitting the data (circles) with Eq. 6, fit curve is shown as line, we extract and quantify the multiphoton coupling strength *g* and the cooperativity $C=\frac{4g^{2}}{\kappa \Gamma_{0}}$ depending on the number of pump photons. The cooperativity extracted from the experimental data is shown as circles in (**D**), and the theoretical curve based on the fit in (C) is shown as a line. The gray-shaded area for *n*_c_ > 0.1 indicates the regime of cooperativity C > 1. Error bars in (D) correspond to a 1-kHz uncertainty in the bare RF linewidth Γ_0_. Here, the best agreement with the data was found with Γ_0_ = 2π · 27.5 kHz.

We characterize the total damping rate of the RF resonator by probing its response $S_{11}^{\text{RF}}$ in reflection with a small probe tone while pumping the HF mode with a variable power microwave tone exactly on the red sideband ω_p_ = Ω_0_ − Ω_0_. The experimental scheme is shown in Fig. 2A, and the result of the response measurement is plotted in Fig. 2B for varying HF sideband pump powers. With increasing HF intracavity photon number *n*_c_, the total linewidth Γ_eff_ of the RF mode increases from about 2π · 30 kHz at low pump powers to ∼2π · 180 kHz for pump powers that correspond to *n*_c_ ∼ 0.4 intracavity microwave photons. From fits to the response data, the effective damping rate for each pump power is extracted; the result is shown in Fig. 2C. The experimental data are fitted with the theoretical expression for the total damping

$$\Gamma_{\text{eff}}=\frac{\kappa +\Gamma_{0}}{2}-\sqrt{\frac{{\left(\kappa -\Gamma_{0}\right)}^{2}}{4}-4g^{2}}$$ (4)

and as fit parameter, we get the multiphoton coupling strength *g* and subsequently the multiphoton cooperativity $C=\frac{4g^{2}}{\kappa \Gamma_{0}}$. The result is shown in Fig. 2D and demonstrates that we reach large values C > 1 for 0.1 pump photon and a single-photon cooperativity $C_{0}=\frac{4g_{0}^{2}}{\kappa \Gamma_{0}}\approx 10$. With the knowledge of *g*, Γ_0_, and κ for a given pump strength, the photon-pressure interaction is fully characterized.

Without the RF probe tone applied in the previous experiment, the currents in the RF mode are given by residual thermal and quantum fluctuations. These current fluctuations lead to resonance frequency fluctuations of the HF mode, mediated by the SQUID. Therefore, when the HF mode is driven with a continuous frequency pump tone on the red sideband, the resonance frequency fluctuations induced by the LF mode lead to the generation of a sideband at ω_p_ + Ω_0_. This sideband corresponds to upconverted thermal photons from the RF mode, and its detection and analysis allow to determine the residual RF mode occupation. The power spectral density at the detector [HF HEMT (High-Electron-Mobility Transistor) amplifier] input in units of quanta for a pump around the red sideband is in good approximation given by

$$\frac{S(\omega )}{\hbar \omega }=\frac{1}{2}+n_{\text{add}}\prime +\frac{\kappa_{e}g^{2}{|\chi_{0}|}^{2}{|\chi_{c}|}^{2}\Gamma_{0}}{{|1+g^{2}\chi_{c}\chi_{0}|}^{2}}n_{\text{th}}^{\text{RF}}$$ (5)

with the RF mode occupation as weighted sum of internal and external bath occupations $n_{\text{th}}^{\text{RF}}=\frac{\Gamma_{e}}{\Gamma_{0}}n_{e}^{\text{RF}}+\frac{\Gamma_{i}}{\Gamma_{0}}n_{i}^{\text{RF}}$ and the HF and RF mode susceptibilities

$$\chi_{c}^{-1}=\frac{\kappa }{2}+i(\omega -\omega_{0})$$ (6)

$$\chi_{0}^{-1}=\frac{\Gamma_{0}}{2}+i(\omega -\omega_{p}-\Omega_{0})$$ (7)

respectively. For Eq. 5, we assumed that internal and external bath of the HF cavity are well equilibrated to the fridge temperature $T_{i}^{\text{HF}},T_{e}^{\text{HF}}\&lt;100$mK which translates to $n_{i}^{\text{HF}},n_{e}^{\text{HF}}\ll n_{\text{th}}^{\text{RF}},{n_{\text{add}}}^{\prime },1/2$; for details, cf. note S6 (*52*).

**Fig. 3.**
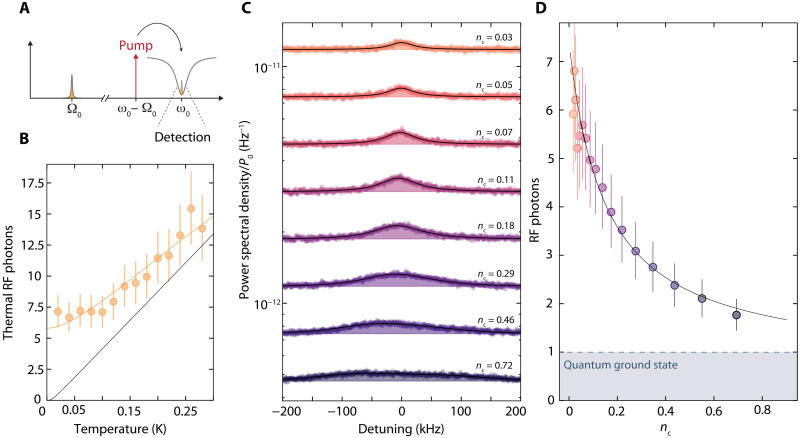
Sideband cooling of a hot RF resonator with less than a single pump photon. (**A**) For the observation of upconverted thermal noise and cooling of the RF resonator, a pump tone is set to the red sideband of the HF mode ω_p_ = ω_0_ − Ω_0_, and the cavity output field around ω = ω_0_ is detected with a signal analyzer. (**B**) Thermal RF photon number versus fridge temperature. Symbols are data, the black line is the Bose factor, and the orange line is a more elaborate model taking into account thermal radiation on the feedline and imperfect chip thermalization; cf. note S7. From this thermal calibration, we determine the thermal occupation of the RF mode at fridge base temperature to be $n_{\text{th}}^{\text{RF}}\sim 7\pm 1$. (**C**) Measured HF output power spectral density for increasing red-sideband pump power normalized to the on-chip pump power P_0_. Frequency axis is given as detuning from the low-power noise center frequency. Circles are data, and lines with shaded areas are fits. With increasing pump strength, the RF resonance gets broadened by photon-pressure damping, and its total thermal noise power gets reduced, which corresponds to sideband cooling of the RF mode. The slight asymmetry in the power spectral density for the largest pump powers originates from a small detuning δ ≈ 2π ∙ 30 kHz of the pump from the red sideband, which is taken into account in our analysis. In (**D**), the thermal mode occupation photon number is shown as symbols versus HF pump photon number. The initial thermal occupation is cooled by about a factor of ~4, and theoretical expectation is shown as a line. Error bars in (B) and (D) correspond to uncertainties of ±2 HF photons of added noise in the detection chain and ± 2 kHz in bare RF linewidth Γ_0_.

From a thermal calibration of the RF mode occupation with varying fridge temperature, shown in Fig. 3B, we determine the residual occupation at base temperature to be ∼7 ± 1 RF photons and the effective number of noise photons added by the detection chain *n*_add_′ ≈ 11 ± 2; details are given in notes S7 and S8. We note that we observe a dependence of the bare linewidth Γ_0_ not only on the temperature of the mixing chamber but also on the residual RF occupation at *T* = *T*_b_, which we attribute to two-level system saturation in the amorphous silicon dielectric filling of the RF parallel-plate capacitor. For the fridge base temperature data in Fig. 3B, we find Γ_0_ ≈ 2π · 40 kHz, an increased value compared to the values obtained from the reflection measurement. This linewidth and $n_{\text{th}}^{\text{RF}}∼7$ correspond to a single-photon quantum cooperativity $C_{q0}=C_{0}/n_{\text{th}}^{\text{RF}}\approx 1$.

The effective temperature of the RF mode occupied with seven thermal photons is *T*^RF^ ≈ 150 mK and thus considerably higher than the base temperature of the mixing chamber *T*_f_ ≈ 15 mK. We attribute this mostly to radiative noise heating through the RF input/output feedline, which is not strongly isolated from the cryogenic RF amplifier mounted in between the 800-mK and the 3.2-K plates, but we can also not completely exclude a small contibution from imperfect thermalization of the device to the mixing chamber. This interpretation is supported by the increase of RF mode occupation when the cryogenic RF amplifier is switched on, in which case we find $n_{\text{th}}^{\text{RF}}∼21$. For the thermal calibration and the cooling experiment presented in Fig. 3, however, the amplifier is switched off. Additional thermal calibration and cooling data with the RF amplifier switched on can be found in note S8.

With the fridge temperature set back to its minimal value *T*_b_ = 15 mK, we measure the HF mode output spectra for varying red-sideband pump power; cf. Fig. 3C. For the smallest pump power shown, the upcoverted thermal noise spectrum displays a Lorentzian lineshape with an effective linewidth Γ_eff_ ≈ 2π · 65 kHz, broadened by dynamical backaction. With increasing sideband pump power, the thermal noise peak broadens further until, for the largest powers, the lineshape deviates from a Lorentzian because of the onset of normal-mode splitting. Additional spectra for a larger residual RF occupation $n_{\text{th}}^{\text{RF}}∼21$ are given in note S8.

By fitting the spectra with Eq. 5, shown as lines and shaded areas in Fig. 3C, the equilibrium RF photon numbers are determined and converted to the sideband-cooled photon occupation

$$n_{\text{cool}}^{\text{RF}}=n_{\text{th}}^{\text{RF}}\frac{\Gamma_{0}}{\kappa +\Gamma_{0}}\frac{4g^{2}+\kappa (\kappa +\Gamma_{0})}{4g^{2}+\kappa \Gamma_{0}}+n_{\text{th}}^{\text{HF}}\frac{\kappa }{\kappa +\Gamma_{0}}\frac{4g^{2}}{4g^{2}+\kappa \Gamma_{0}}$$ (8)

Note that this relation differs from the result usually quoted in optomechanics (*1*, *20*, *53*), which is only valid for κ ≫ Γ_0_ and underestimates the cooling rate in the unusual regime Γ_0_ ≲ κ. The full equation taking into account also finite pump detunings δ can be found at the end of note S6.

The resulting sideband-cooled RF mode occupation is shown in Fig. 3D. Therefore, with less than a single pump photon, the RF mode is cooled by about a factor of ∼4 to an occupation of only 1.7 RF quanta, demonstrating the applicability of sideband cooling for photon-pressure–coupled circuits and an extraordinarily large single-photon cooling rate. At higher pump powers than the ones discussed so far, the two circuits hybridize in the parametric normal-mode splitting regime (*20*, *26*, *53*, *54*).

In the strong-coupling regime, i.e., when the frequency splitting of the normal modes exceeds the hybridized linewidths, the residual thermal occupation of the RF mode is distributed between the hybridized normal modes. Equation 8, however, remains valid, and the onset of mode hybridization does not prevent the RF mode from being cooled further. The theoretical limit for cooling in the regime *g* ≫ κ, Γ_0_ is given by $n_{\text{lim}}^{\text{RF}}=n_{\text{th}}^{\text{RF}}\Gamma_{0}/(\kappa +\Gamma_{0})\approx 0.67$ photons, assuming a ground state HF cavity. The remaining thermal excitations of the system are then equally distributed between the RF and the HF modes; cf. also note S9.

**Fig. 4.**
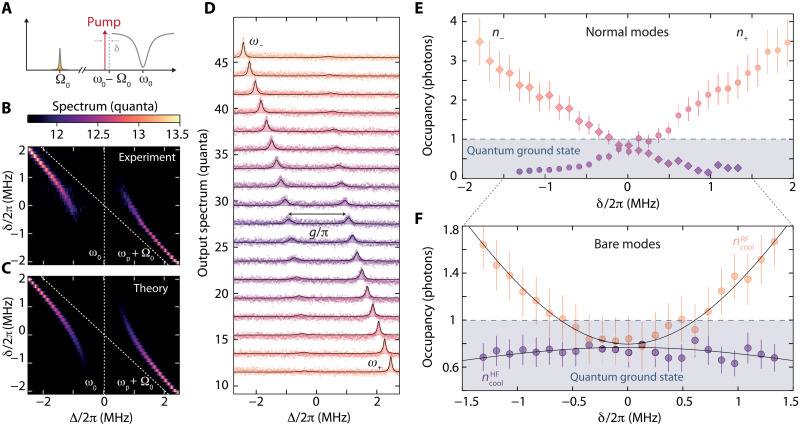
Normal-mode thermometry and ground state cooling in the quantum coherent strong-coupling regime. (**A**) Experimental scheme. A strong pump tone is applied with detuning δ = ω_p_ − (ω_0_ − Ω_0_) from the red sideband of the HF mode. For each pump detuning δ, the output spectrum around the bare HF mode resonance frequency ω_0_ + Δ is measured. The resulting power spectra are plotted color-coded in (**B**). (**C**) Result of calculations based on the theoretical model. In (**D**), we plot the corresponding linecuts from (B) and (C) on top of each other, displaying a high level of agreement between experiment and theory. The bottom data are displayed with an unmodified background level of 11.5, and subsequent lines are offset by +2 quanta each. In the regime δ = 0, the splitting between the modes is given by *g*/π with *g* = 2π∙1 MHz. We treat the two parametrically coupled normal modes as individual HF modes with resonance frequencies ω_−_ and ω_+_ and extract the corresponding, effective thermal photon numbers n_−_ and n_+_ from the spectrum shown in (D). The values are plotted in (**E**). From the normal-mode occupations *n*±, we determine the occupation of the bare HF and RF modes shown in (**F**). Around δ = 0, both bare modes are in the quantum ground state with residual occupations $n_{\text{cool}}^{\text{HF}}\approx n_{\text{cool}}^{\text{RF}}∼0.8\pm 0.2$. For the theoretical calculations, shown as lines, we assume $n_{\text{th}}^{\text{HF}}=0.01$ and find as equilibrium thermal occupations $n_{\text{th}}^{\text{HF}}=8.0$. Error bars in (E) and (F) correspond to uncertainties of ±2 HF photons of added noise in the detection chain and ± 2 kHz in Γ_0_.

To characterize the residual number of thermal RF photons in the strong-coupling regime, we detect the output noise of the normal modes in the HF domain for varying detuning of the pump tone from the red sideband δ. In Fig. 4, the measured output spectra around Δ = ω − ω_0_ are shown color-coded in Fig. 4B and as individual line scans in Fig. 4D. We find an excellent agreement between the data and theoretical calculations, shown color-coded in Fig. 4C and as lines in Fig. 4D. For large detunings ∣δ∣ > 2π · 2 MHz, a hot mode with a large noise amplitude is observed, whose frequency follows closely ω_p_ + Ω_0_ and corresponds to the normal mode dominated by the RF circuit in this regime. At the same time, no output noise field is detected around the HF cavity–like normal mode close to Δ = 0, indicating that this mode is cold and in thermal equilibrium with its bath. For small detunings δ ∼ 0, we observe a pronounced avoided crossing of the RF mode with the driven HF cavity, centered at Δ = 0. The splitting between the two hybridized modes is given by *g*/π ≈ 2 MHz, indicating that we reach the so-called quantum-coherent coupling regime where $g\&gt;\kappa ,\Gamma_{0}n_{\text{th}}^{\text{RF}}$ (*45*) and a quantum cooperativity $C_{q}=C/n_{\text{th}}^{\text{RF}}\approx 35$.

For a quantification of the effective normal-mode thermal occupation, we treat the modes as two independent HF modes; a detailed description is given in notes S9 and S10. The lower-frequency mode has the resonance frequency ω_−_, linewidth κ_−_, and external linewidth κ_e−_, the higher-frequency mode ω_+_, κ_+_, and κ_e+_, respectively. The complex resonances of the normal modes are given by (*53*)

$${\tilde{\omega }}_{\pm }=\omega_{0}+\frac{\delta }{2}+i\frac{\kappa +\Gamma_{0}}{4}\pm \sqrt{g^{2}-{\left(\frac{\kappa -\Gamma_{0}+2i\delta }{4}\right)}^{2}}$$ (9)

and the resonance frequencies and linewidths are $\omega_{\pm }=\text{Re}[{\tilde{\omega }}_{\pm }]$ and $\kappa_{\pm }=2\text{Im}[{\tilde{\omega }}_{\pm }]$, respectively.

The power spectral density of the HF output field in terms of these normal-mode parameters can be written as

$$\frac{S_{\text{nms}}}{\hbar \omega }=\frac{1}{2}+{n_{\text{add}}}^{\prime }+4\frac{\kappa_{e-}\kappa_{-}}{\kappa_{-}^{2}+4\Delta_{-}^{2}}n_{-}+4\frac{\kappa_{e+}\kappa_{+}}{\kappa_{+}^{2}+4\Delta_{+}^{2}}n_{+}$$ (10)

where *n*_±_ are the effective photon occupations of the two normal modes, Δ_±_ = ω − ω_±_ and the external linewidths $\kappa_{e\pm }=\frac{\kappa_{e}}{2}(1\pm \frac{\delta }{\sqrt{\delta^{2}+4g^{2}}})$; cf. notes S9 and S10. The effective normal-mode occupations *n*_−_ and *n*_+_ depending on the pump detuning are shown in Fig. 4E. While for large detunings the RF-like normal mode is still hot and the HF-like mode is in the quantum ground state, both normal modes appear to be in the quantum ground state when they are close to the full-mode hybridizaton at δ ∼ 0. The minimum occupation that we observe at the symmetry point is *n*_−_ = *n*_+_ = 0.8.

From comparison between the two versions of the HF power spectral densities (Eqs. 10 and 5) and the condition *S*_nms_ = *S*, we obtain the HF mode occupation in the cooling regime $n_{\text{cool}}^{\text{HF}}$ by

$$\kappa_{e}n_{\text{cool}}^{\text{HF}}=\kappa_{e-}n_{-}+\kappa_{e+}n_{+}$$ (11)

For zero pump detuning, this simplifies to $n_{\text{cool}}^{\text{HF}}=n_{-}=n_{+}=0.8$. Using the equation for the total thermal occupation $n_{\text{cool}}^{\text{tot}}$ of the system in the strong-coupling regime (cf. note S9), we calculate the residual occupation of the RF mode $n_{\text{cool}}^{\text{RF}}=n_{\text{cool}}^{tot}-n_{\text{cool}}^{\text{HF}}$. The resulting occupation of both bare modes is shown in Fig. 4F, showing that $n_{\text{cool}}^{\text{RF}}\approx n_{\text{cool}}^{\text{HF}}=0.8\pm 0.2$, i.e., both are in the quantum ground state for δ ∼ 0.

## DISCUSSION

In future devices, both the cooling factor and the thermal noise detection efficiency could be considerably improved by reducing the external linewidth of the HF cavity by one to two orders of magnitude. A correspondingly optimized device might also be suitable to generate quantum-squeezed RF states or to entangle distinct RF circuits, similar to what has been reported for optomechanical devices (*3*, *7*). If at the same time the HF cavity flux noise can be reduced by, for example, improved shielding and/or lower-noise current sources, then an HF cavity operation point with larger *g*_0_ can be chosen while maintaining the high values for the single-photon cooperativity reported in the current work.

With the results presented in this work, we demonstrated photon-pressure coupling of a hot RF circuit to a superconducting microwave cavity in the quantum regime. By a galvanically connected circuit design, we markedly increased the single-photon coupling strength and achieved a single-photon quantum cooperativity of unity. On the basis of the large single-photon coupling rate, we were able to demonstrate both sideband cooling of the RF mode by a factor of 4 and the strong-coupling regime, with less than a single pump photon. For stronger pump powers, we enter the quantum-coherent coupling regime and demonstrate photon-pressure ground state cooling of the originally hot RF mode.

Compared to other recently developed radiative cooling techniques of circuits and other systems (*55*, *56*), sideband cooling can reduce the effective mode temperature far below the physical temperature of any bath (*57*). Furthermore, in contrast to previous reports of sideband-cooling techniques with circuits using highly nonlinear systems such as superconducting qubits (*58*, *59*), our approach allows for both participating circuits to have a very high degree of linearity which is highly desirably for many signal processing applications. This work lays the foundation for RF quantum photonics for quantum-limited RF sensing and has potential applications in quantum-limited microwave signal and bosonic code quantum information processing based on photon-pressure–coupled circuits.

## MATERIALS AND METHODS

The photon-pressure system used in this experiment consists of two galvanically connected lumped-element *LC* circuits, and it was engineered via a multilayer nanofabrication process. The HF mode of the circuit comprises two interdigitated capacitors, a SQUID containing two Josephson junctions and two linear inductors. Aside from the firstly patterned 50-nm-wide, 100-nm-long, and 15-nm-thick aluminum nanobridge junctions and their respective 500 nm by 500 nm contact pads, the circuit is made of a ∼70-nm-thick aluminum layer on a silicon substrate. Both layers were patterned via a sputtering deposition in combination with electron beam lithography and liftoff. In addition, an argon milling process (∼2 min) was performed in situ before the second deposition step to provide good electrical contact between the two layers. The RF mode is formed by a parallel-plate capacitor with a ∼130-nm-thick amorphous silicon layer as a dielectric and a short inductor wire that simultaneously acts as the loop of the SQUID. The inductor wire was patterned together with the bottom capacitor plate and the HF circuit mode components. Subsequent to this step, the dielectric deposition took place, i.e., a PECVD (plasma-enhanced chemical vapor deposition) process followed by a reactive ion etching step and O_2_ plasma ashing. Last, we patterned the top capacitor plate. This one is made of a 250-nm aluminum layer, and it was fabricated via another sputtering-liftoff procedure, which once again included an in situ argon milling to guarantee good contact between the plates. A step-by-step description of the device fabrication is given in note S1.

## Supplementary Materials

### This PDF file includes:

Notes S1 to S10 Figs. S1 to S12
