# Supplementary material for: Cooling photon-pressure circuits into the quantum regime
Source: Sci Adv. 2021 Oct 15;7(42):eabg6653. doi: 10.1126/sciadv.abg6653 (PMC8519572; doi:10.1126/sciadv.abg6653)
Supplement: Supplementary file 1 — Notes S1 to S10 Figs. S1 to S12 [file sciadv.abg6653_sm.pdf]

Science Advances
AAAS

Supplementary Materials for

Cooling photon-pressure circuits into the quantum regime

Ines Corveira Rodrigues*, Daniel Bothner, Gary Alexander Steele

*Corresponding author. Email: i.c.corveirarodrigues@tudelft.nl

Published 15 October 2021, *Sci. Adv.* **7**, eabg6653 (2021)
DOI: 10.1126/sciadv.abg6653

This PDF file includes:

Notes S1 to S10
Figs. S1 to S12

SUPPLEMENTARY NOTE 1: DEVICE FABRICATION

- **Step 0: Marker patterning.** Prior to the device fabrication, we perform the patterning of alignment markers on a full 4 inch Silicon wafer, required for the electron-beam lithography (EBL) alignment of the following fabrication steps. The structures were patterned using a CSAR62.13 resist mask and sputter deposition of 50 nm Molybdenum-Rhenium alloy. After undergoing a lift-off process, the only remaining structures on the wafer were the markers. The complete wafer was diced into $14 \times 14\,\mathrm{mm}^2$ chips, which were used individually for the subsequent fabrication steps. The step was finalized by a series of several acetone and IPA rinses.

- **Step 1: Junctions patterning.** As first step in the fabrication, we pattern weak links which afterwards result in constriction type Josephson junctions between the arms of the SQUID. The weak link nanowires were patterned together with larger pads, cf. Supplementary Fig. 1**a**, which were used to achieve good electrical contact with the rest of the circuit, cf. Step 3. The nanowires are designed to be $\sim 50\,\mathrm{nm}$ wide and $\sim 100\,\mathrm{nm}$ long at this point of the fabrication, and each pad is $500 \times 500\,\mathrm{nm}^2$ large. For this fabrication step, a CSAR62.09 was used as EBL resist and the development was done by dipping the exposed sample into Pentylacetate for 60 seconds, followed by a solution of MIBK:IPA (1:1) for 60 seconds, and finally rinsed in IPA, where MIBK is short for methyl isobutyl ketone and IPA for isopropyl alcohol. The sample was subsequently loaded into a sputtering machine where a 15 nm layer of Aluminum was deposited. Finally, the chip was placed at the bottom of a beaker containing a small amount of Anisole and inserted into an ultrasonic bath for a few minutes where the sample underwent a lift-off process. The step was finalized by a series of several acetone and IPA rinses.

- **Step 2: Bottom RF capacitor plate and HF resonator patterning.** As second step in the fabrication, we pattern the bottom plate of the parallel plate capacitor, the inductor wire of the radio-frequency cavity, which also forms part of the SQUID loop, the remaining part of the SQUID cavity (cf. Supplementary Fig. 1**b**.) and the center conductor of the SQUID cavity feedline by means of EBL using CSAR62.13 as resist. After the exposure, the sample was developed in the same way as in the first fabrication step and loaded into a sputtering machine. In the sputter system, we performed an argon milling step for two minutes and afterwards deposited 70 nm of Aluminum. The milling step, performed in-situ and prior to the deposition, very efficiently removes the oxide layer which was formed on top of the previously sputtered weak link pads, and therefore allows for good electrical contact between the two layers. After the deposition, the unpatterned area was lifted-off by means of an ultrasonic bath in room-temperature Anisole for a few minutes. The step was finalized by a series of several acetone and IPA rinses.

- **Step 3: Amorphous silicon deposition.** The deposition of the dielectric layer of the parallel plate capacitor was done using a plasma-enhanced chemical vapor deposition (PECVD) process. To guarantee low dielectric losses in the material, the chamber underwent an RF cleaning process overnight and only afterwards the deposition of $\sim 130\,\mathrm{nm}$ of amorphous silicon was performed. At this point of the fabrication, the whole sample is covered with dielectric, cf. Supplementary Fig. 1**c**.

- **Step 4: Reactive ion etch patterning of αSi.** We spin-coat a double layer of resist (PMMA 950K A4 and ARN-7700-18) on top of the αSi-covered sample, and expose the next pattern with EBL. Prior to the development of the pattern, a post-bake of 2 minutes at $\sim 115\,^\circ\mathrm{C}$ was required. Directly after, the sample was dipped into MF-321 developer for 2 minutes and 30 seconds, followed by H_2O for 30 seconds and lastly rinsed in IPA. To finish the third step of the fabrication, the developed sample underwent a SF_6/He reactive ion etching (RIE) to remove the amorphous Silicon. To conclude the etching step, we performed a O_2 plasma ashing in-situ with the RIE process to remove resist residues, the result is shown schematically in Supplementary Fig. 1**d**.

- **Step 5: Top capacitor plate and ground-plane patterning.** As final step, the sample was again coated in CSAR62.13 and the top plate of the RF capacitor as well as all ground plane and the low-frequency feedline was patterned with EBL. The resist development was done identical to the ones in the second and third steps. Afterwards, the sample was loaded into a sputtering machine where an argon milling process was performed in-situ for 2 minutes, in order to have good electrical contact between the top and bottom plates of the low-frequency capacitor, similar to what was done between the second and third fabrication steps. After the milling, a 250 nm layer of Aluminum was deposited and finally an ultrasonic lift-off procedure was performed. The step was finalized by a series of several acetone and IPA rinses. With this, the sample fabrication process was essentially completed, cf. Supplementary Fig. 1**e**.

- **Step 6: Dicing and mounting.** At the end of the fabrication, the sample was diced to a $10 \times 10\,\mathrm{mm}^2$ size and mounted to a printed circuit board (PCB), wire-bonded to microwave feedlines and ground and packaged into a radiation tight copper housing.

A schematic representation of this fabrication process can be seen in Supplementary Fig. 1, omitting the initial patterning of the electron beam markers and the sample mounting. In addition, an optical image of the full device is shown in Supplementary Fig. 2.

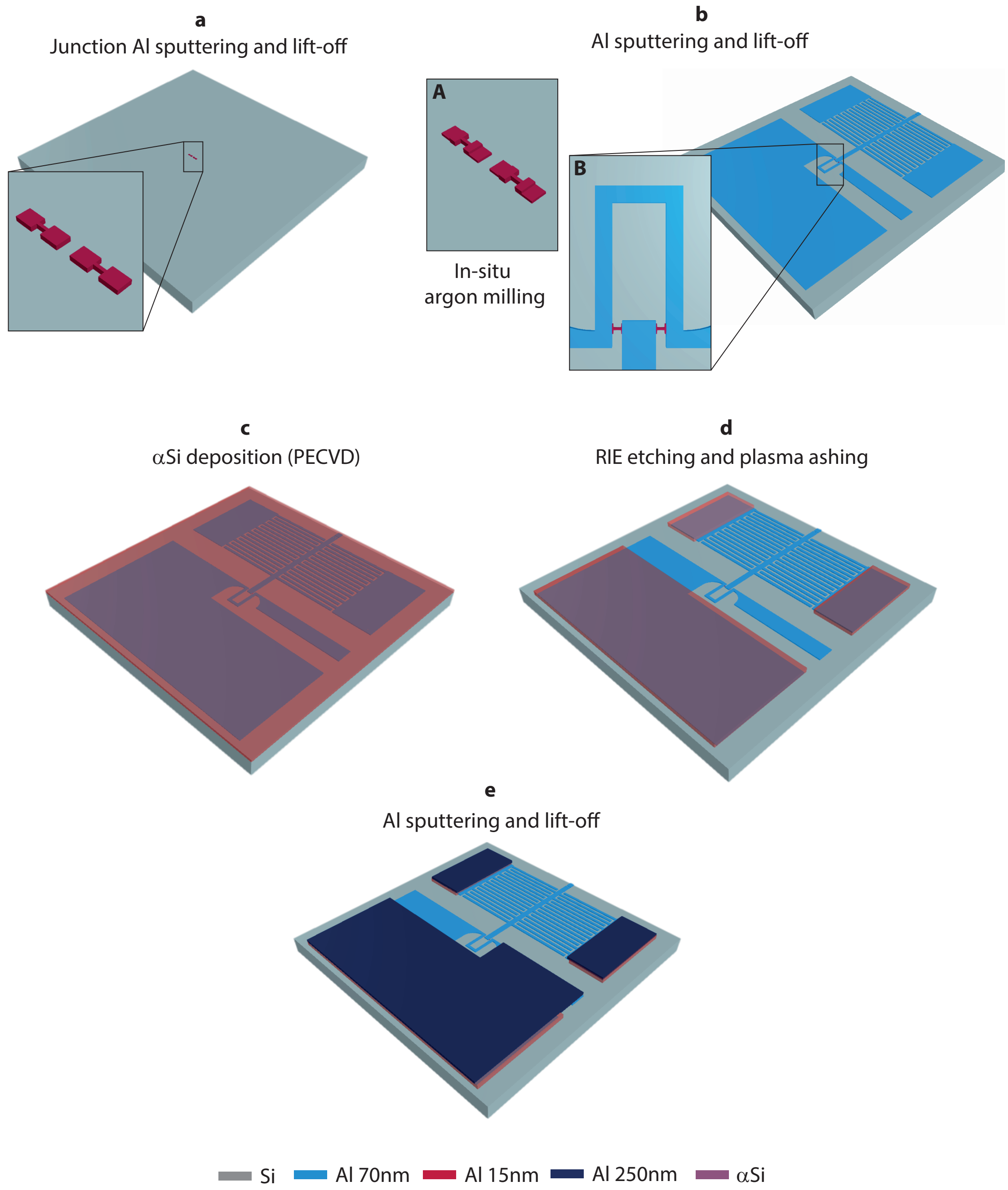

Supplementary Figure 1. **Schematic device fabrication.** **a** shows the weak-link Josephson junctions with contact pads, patterned in the first fabrication step. **b** shows the patterned second Aluminum layer, forming the bottom of the RF parallel plate capacitor, the SQUID loop and the HF cavity. Inset **A** showing the in-situ argon milled Josephson junctions prior to the deposition (the existing resist is not shown for better visibility of the milled structures). Inset **B** shows a zoom-in of the 3D SQUID. **c** shows the sample after the deposition of αSi. **d** shows the device after the subsequent SF_6/He reactive ion etching step, finished by an in-situ O_2 plasma ashing. **e** shows the final device after the deposition of the last Aluminum layer.

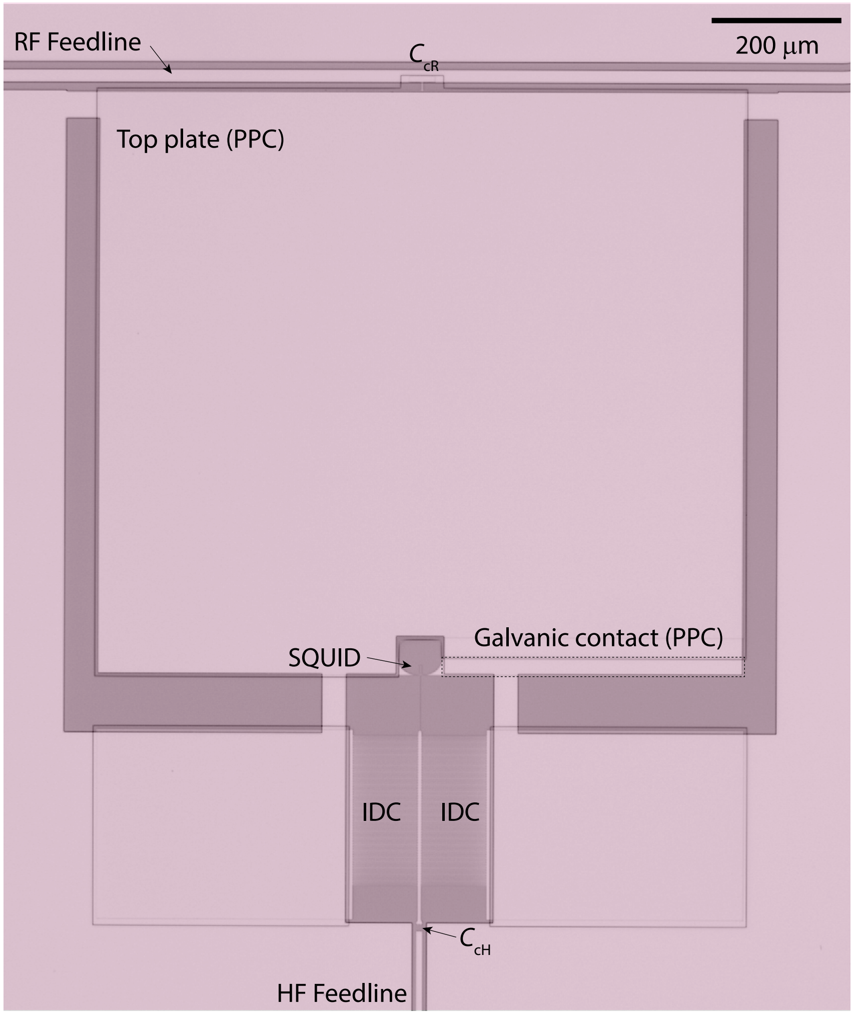

Supplementary Figure 2. **Optical image of the full device.** Visible are both, the radio-frequency (top) and microwave (bottom) resonators including their corresponding RF and HF feedlines. The coupling capacitors are labelled with C_{cR} for the RF mode and C_{cH} for the HF mode, respectively. Also labelled are the RF parallel plate capacitor (PPC), the HF interdigitated capacitors (IDC), and the SQUID for orientation. Zoom-ins to the HF mode circuit including labels for the inductors are shown in main paper Fig. 1. The galvanic contact area of the PPC top and bottom plates is marked with a dashed rectangle.

SUPPLEMENTARY NOTE 2: MEASUREMENT SETUP

All the experiments reported in this paper were performed in a dilution refrigerator operating at a base temperature close to $T_{\mathrm{b}} = 15\,\mathrm{mK}$. A schematic of the experimental setup and of the external configurations used in the different performed experiments can be seen in Supplementary Fig. 3.

The printed circuit board (PCB), onto which the fabricated sample was glued and wire-bonded, was placed in a radiation tight copper housing and connected to two coaxial lines. One of the lines was used as input/output port for the high-frequency (HF) SQUID cavity and the second line was set in a similar way for the radio-frequency resonator. Both of the cavities were measured in a reflection geometry, and therefore the input and output signals were split via a directional coupler. For the HF line, the directional coupler was positioned on the 15 mK stage, while for the RF line it was mounted in between the 15 mK plate and the 100 mK plate. Both output signals went into a cryogenic amplifier for their particular frequency range.

Furthermore, in order to generate an out-of-plane magnetic field, required to flux bias the SQUID cavity, an external magnet (not shown in the figure) was put in very close proximity below the device and the two were placed inside a

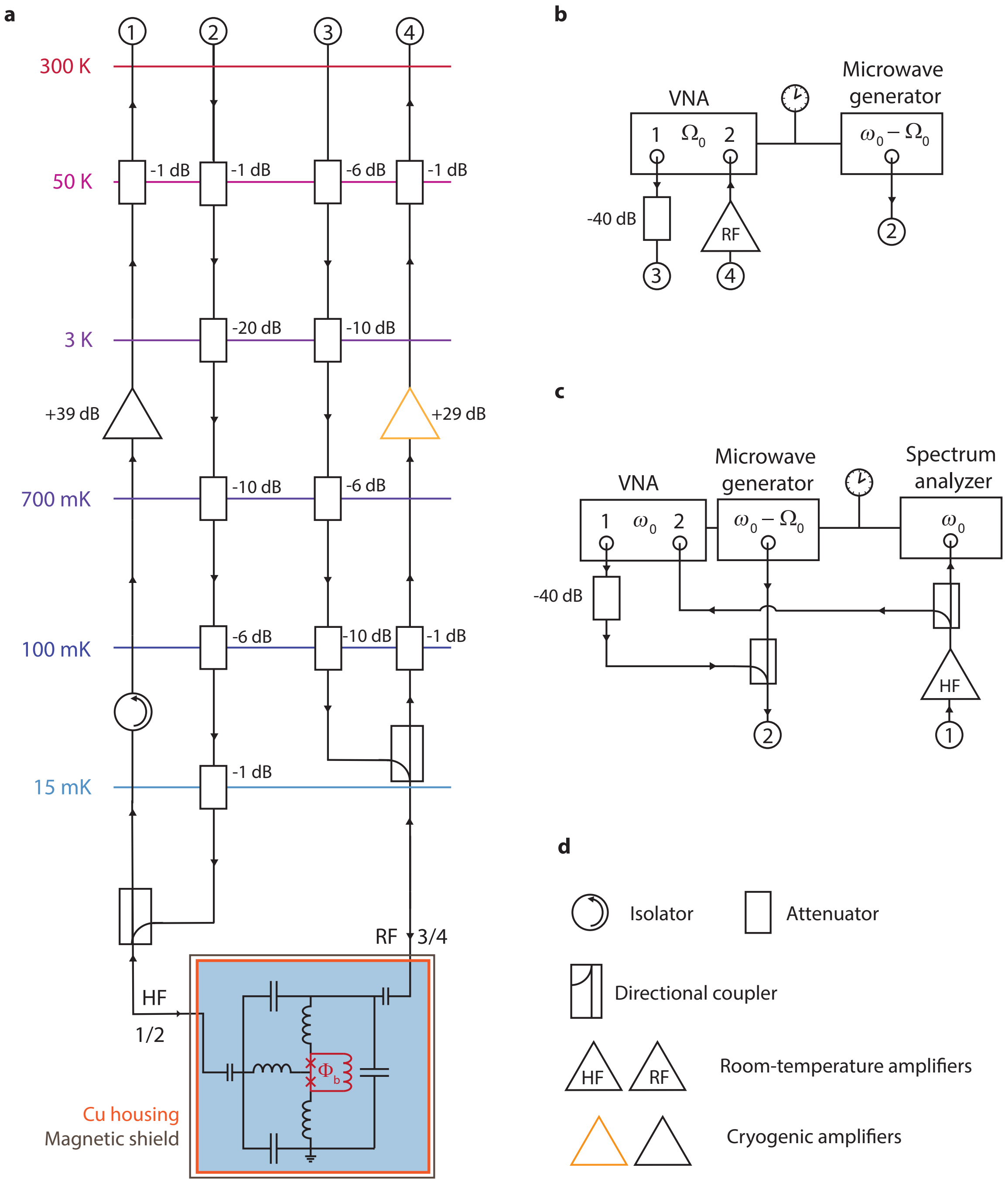

Supplementary Figure 3. **Schematic of the measurement setup.** Detailed information is provided in text.

cryoperm magnetic shield. The magnet was connected with DC wires, allowing for the field to be tuned by means of a DC current (not shown).

Both input lines were heavily attenuated in order to balance the thermal radiation from the line to the base temperature of the fridge. The low-frequency line, however, is not fully equilibrated to the fridge base temperature due to the lack of cryogenic circulators/isolators for the particular frequency range. Outside of the refrigerator, we used different configurations of microwave signal sources and high-frequency electronics for the different experiments.

In **b** we show the configuration used to measure the photon-pressure damping of the radio-frequency mode (main paper Fig. 2). A microwave generator sends a continuous wave signal to the SQUID cavity around its red sideband,

while the RF resonator is probed in reflection with a vector network analyzer (VNA).
In **c** we show the setup for photon-pressure sideband-cooling experiment and the normal-mode thermometry (main paper Figs. 3 and 4), where a continuous wave tone is send to the red sideband of the SQUID cavity. In addition, in order to observe the cavity response and adjust the pump tone frequency with respect to the power-dependent cavity resonance, a weak VNA signal is combined with the pump tone via a directional coupler. The output signal is analyzed individually by a spectrum analyzer and a VNA after being amplified. During the detection of thermal noise with the signal analyzer, the VNA scan was stopped and the VNA output power was completely switched off.
For the normal-mode thermometry experiment, we replaced the 20 dB attenuator on the 3 K plate of HF input line 2 by a 10 dB one. This allowed for stronger red-sideband pumping and therefore for reaching deeper into the strong-coupling regime.
For all experiments, the microwave sources and vector network analyzers (VNA) as well as the spectrum analyzer used a single reference clock of one of the devices.

Power Calibration

In order to estimate the input power on the on-chip high-frequency feedline of the device, we use the thermal noise of the HF HEMT (High-Electron-Mobility Transistor) amplifier as calibration method. The cryogenic HEMT amplifier thermal noise power is given by

$$P_{\mathrm{HEMT}} = 10\,\log\left(\frac{k_{\mathrm{B}} T_{\mathrm{HEMT}} \Delta f}{1\,\mathrm{mW}}\right) \tag{1}$$

where k_{B} is the Boltzmann constant, T_{HEMT} is the noise temperature of the amplifier, which, according to the specification datasheet, is approximately 2 K, and $\Delta f = 2000\,\mathrm{Hz}$ is the measurement IF bandwidth. The calculated noise power is $P_{\mathrm{HEMT}} = -162.58\,\mathrm{dBm}$, or as noise RMS voltage $\Delta V = 1.66\,\mathrm{nV}$.

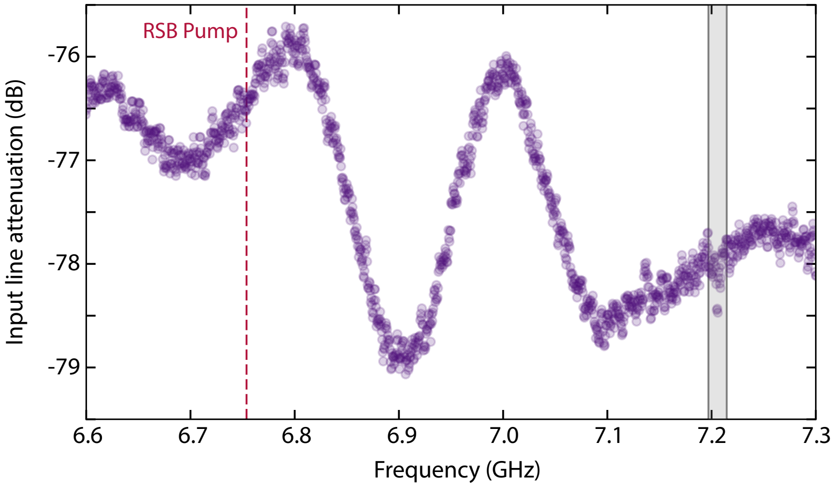

Supplementary Figure 4. **Estimation of the frequency-dependent input line attenuation for the pump tone.** The data are obtained by measuring 501 traces in the shown frequency range using the vector network analyzer, cf. Supplementary Fig. 3. For each frequency point, we determine from the 501 traces the signal-to-noise ratio and with the assumption of a frequency-independent HEMT noise temperature and 2.7 dB losses between the sample and the HEMT, we get the input line attenuation as plotted. The gray area shows where the cavity was during the calibration. The frequency of the red sideband (RSB) pump tone utilized for the experiments reported in this work is indicated by the red dashed line. The input line attenuation estimated for that point is ~ 76.5 dB.

Taking into account the room temperature attenuators of 40 dB as well as the directional coupler (20 dB loss on the coupled port) for the VNA tone and assuming an attenuation between the sample and the HEMT of 2.7 dB (based on the effective added noise obtained from the thermal calibration, see Supplementary Note 7), we extract a frequency-dependent input line attenuation as shown in Supplementary Fig. 4. The input line attenuation for the frequency of our red sideband pump is ~ 76.5 dB. The deviation between this value and one extracted from the effects of photon-pressure dynamical backaction (cf. Fig. 2 of the main paper) is as small as 0.3 dB.

SUPPLEMENTARY NOTE 3: THE CIRCUIT MODEL AND FLUX DEPENDENCE

The circuit model

The diagram shown in Supplementary Fig. 5 represents the full circuit model of the device. Similar to the simplified version shown in Fig. 1**a** of the main paper, it contains the high-frequency (drawn in purple) and a radio-frequency (drawn in orange) mode, which share the center part of the circuit (drawn in gray). The shared part contains a non-linear, flux-tunable SQUID inductance. The Josephson junctions which form part of the tunable SQUID are constriction type Josephson junctions, which are known to have a current-phase relation (CPR) that can differ significantly from the typical sinusoidal CPR [49, 50]. To include this effect in the flux dependence of the modes, we model each weak-link inductance to be a series combination of a non-linear element L_j with a sinusoidal current-phase relation and a linear inductor L_a.

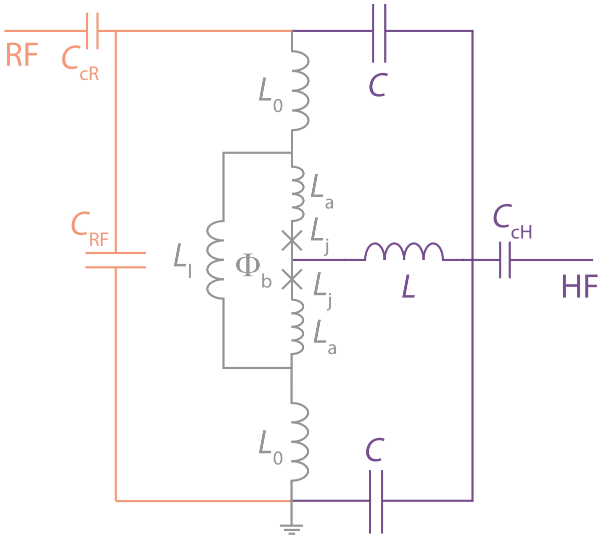

Supplementary Figure 5. **Full circuit diagram of the device.** The orange part of the circuit represents the components belonging only to the radio-frequency mode. The circuit parts drawn in purple correspond to the high-frequency mode. Both modes share the part of the circuit drawn in gray.

Flux dependence of the HF mode

The resonance frequency of a SQUID cavity with a symmetric SQUID can be described by

$$\omega_0(\Phi_\mathrm{b}) = \frac{\omega_0(0)}{\sqrt{\Lambda + \frac{1-\Lambda}{\cos\left(\pi\frac{\Phi}{\Phi_0}\right)}}} \tag{2}$$

where Φ corresponds to the total flux threading the SQUID loop and $\omega_0(0)$ is the resonance frequency without external flux bias (sweetspot frequency). The parameter $\Lambda = (L_\mathrm{HF} - \frac{1}{2}L_\mathrm{j0})/L_\mathrm{HF}$ with the total high-frequency inductance L_HF and the single junction Josephson inductance L_j0 is a measure for the contribution of the Josephson inductance to the total inductance. For zero bias current and the (magnetic plus kinetic) loop inductance $L_\mathrm{loop} = L_\mathrm{l} + 2L_\mathrm{a}$ the total flux threading the SQUID is given by

$$\frac{\Phi}{\Phi_0} = \frac{\Phi_\mathrm{b}}{\Phi_0} + \frac{L_\mathrm{loop}J}{\Phi_0} \tag{3}$$

with the circulating current J. In the absence of a bias current and symmetric junctions, the circulating current is given by

$$J = -I_\mathrm{c}\sin\left(\pi\frac{\Phi}{\Phi_0}\right) \tag{4}$$

with the zero bias critical current of a single junction $I_c = \frac{\Phi_0}{2\pi L_{j0}}$. Using the screening parameter $\beta_L = \frac{2L_{loop}I_c}{\Phi_0} = \frac{L_{loop}}{\pi L_{j0}}$ the relation for the total flux can be written as

$$\frac{\Phi}{\Phi_0} = \frac{\Phi_b}{\Phi_0} - \frac{\beta_L}{2}\sin\left(\pi\frac{\Phi}{\Phi_0}\right). \tag{5}$$

Figure 1**e** of the main paper shows the experimentally determined SQUID cavity resonance frequency modulating with external magnetic flux Φ_b and a fit curve using Eq. 2, where the relation between the applied external flux Φ_b and the total flux in the SQUID Φ is given by Eq. (5). As fit parameters we obtain $\beta_L = 1.07$ and $\Lambda = 0.946$, i.e., the SQUID Josephson inductance contributes about 5.4% to the total HF inductance. Furthermore, we estimate the capacitance of the SQUID cavity $C_{HF} = 2C + C_{cH}$ with the expressions given in Ref. [51] to be ~ 1.3 pF. Based on the sweetspot frequency of the SQUID cavity

$$\omega_0 = \frac{1}{\sqrt{L_{HF}(2C + C_{cH})}}, \tag{6}$$

we extract the total inductance of the high frequency mode to be $L_{HF} = 370$ pH and with Λ we get the inductance of a single junction $L_{j0} = 40$ pH. This inductance corresponds to a critical junction current $I_c = \frac{\Phi_0}{2\pi L_{jc}} \approx 8.3\,\mu$A.
From the screening parameter $\beta_L = 1.07$ and the single-junction inductance $L_{j0} = 40$ pH, we get a loop inductance $L_{loop} = 2L_a + L_l = \pi\beta_L L_{j0} \approx 134$ pH.

Flux dependence of the RF mode

Based on the circuit diagram shown in Supplementary Fig. 5, we find the total inductance of the radio-frequency mode L_{RF} as

$$L_{RF} = 2L_0 + \frac{2(L_j + L_a)L_l}{2(L_j + L_a) + L_l}, \tag{7}$$

with the Josephson inductance of a single junction $L_j = \frac{L_{j0}}{\cos\left(\pi\frac{\Phi}{\Phi_0}\right)}$ and the SQUID loop inductance $L_{loop} = 2L_a + L_l \approx 134$ pH as boundary condition for L_l and L_a. In addition, we independently estimate the parallel plate capacitance $C_{RF} = 659.7$ pF and the parallel plate coupling capacitance $C_{cR} = 0.3$ pF. From the total capacitance $C_{tot} = C_{RF} + C_{cR}$ and the resonance frequency Ω_0, we determine the total inductance of the RF resonator as $L_{RF} \sim 188$ pH.
As the total inductance of the radio-frequency mode is partly composed by the field-dependent Josephson inductance L_j, the resonator resonance frequency will as well modulate with applied magnetic flux Φ_b as

$$\Omega_0 = \frac{1}{\sqrt{C_{tot}\left(2L_0 + L_l\left(1 + \frac{L_l}{2}\frac{\cos\left(\pi\frac{\Phi}{\Phi_0}\right)}{L_{j0} + L_a\cos\left(\pi\frac{\Phi}{\Phi_0}\right)}\right)^{-1}\right)}}. \tag{8}$$

where the relation between Φ and Φ_b is again given by Eq. (5). Supplementary Fig. 6 shows the RF resonance frequency depending on the applied magnetic flux together with a fit curve using Eq. (8), where the parameter $\beta_L = 1.07$ was kept constant as determined from the HF mode flux dependence. From the fit, we extract the parameters $L_a = 43.5$ pH and $L_l = 47$ pH. Based on the returned fit parameters and on Eq. (8), we obtain $L_0 = 75$ pH.
We note here, that without the linear junction inductances L_a, it is not possible to fit both flux dependences with a single set of reasonable parameters.

Flux dependence of the decay rates κ and Γ_0

As the external magnetic field is kept at a non-zero value during the experiment, it is of interest to analyze how the applied magnetic flux Φ_b affects the linewidths of the circuit. For that, we extract the decay rates of both modes κ and Γ_0 while changing the flux bias point. The result is shown in Supplementary Fig. 7 for the positive tuning range. Both linewidths clearly show a strong dependence for values larger than $\sim 0.7\Phi_0$ to $\sim 0.8\Phi_0$. For the operating point used here of $\Phi_b/\Phi_0 = 0.54$, however, they are nearly unmodified compared to the sweetspot values.

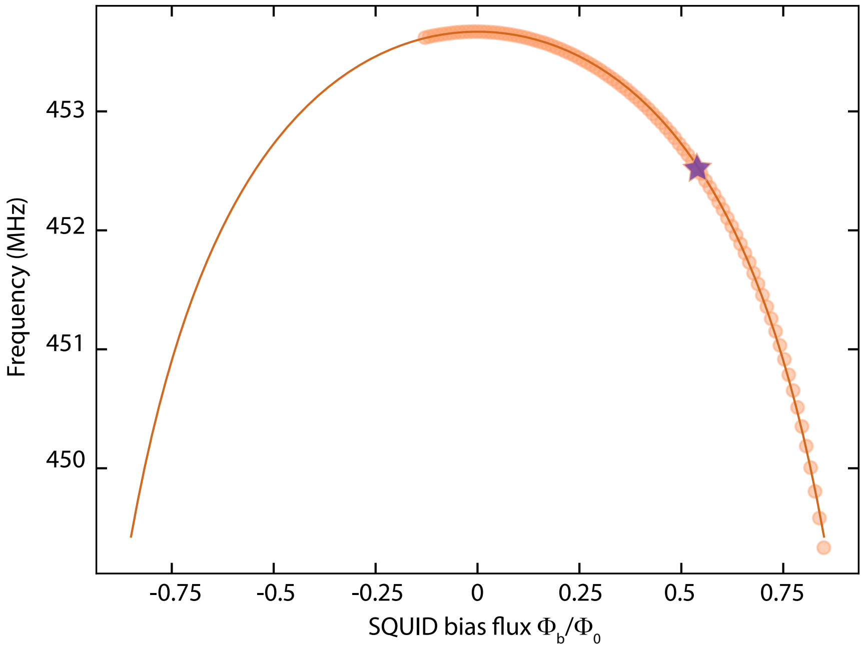

Supplementary Figure 6. **RF mode resonance frequency depending on external magnetic flux.** Points are data and line is a fit curve using Eq. (8). The operation point in this work $\Phi_{\mathrm{b}}/\Phi_0 = 0.54$ is marked by a star.

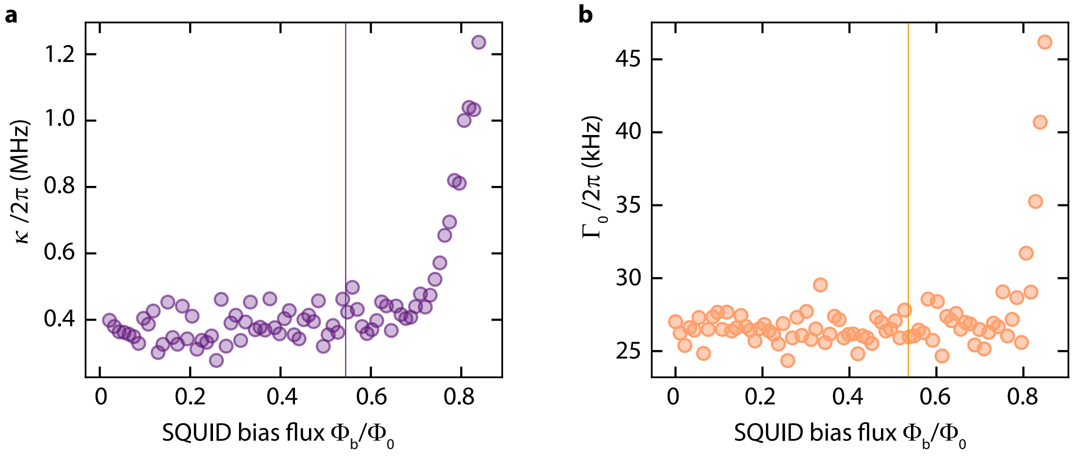

Supplementary Figure 7. **Linewidths for varying SQUID bias flux.** Decay rate of the microwave mode κ (**a**) and of the radio-frequency mode Γ_0 (**b**) depending on magnetic bias flux in units of flux quanta Φ_0. The operation point $\Phi_{\mathrm{b}}/\Phi_0 \approx 0.54$ for the experiments reported here is marked by vertical lines.

SUPPLEMENTARY NOTE 4: RESPONSE FUNCTIONS AND FITTING ROUTINE

Ideal HF and RF resonators response functions

Both, our HF SQUID cavity and the RF resonator, can be modeled as a parallel LC circuit capacitively coupled to a transmission line in a reflection geometry. The S_{11} response function of such a circuit (here for the HF mode) is given by

$$S_{11}^{\mathrm{HF}} = 1 - \frac{2\kappa_{\mathrm{e}}}{\kappa_{\mathrm{i}} + \kappa_{\mathrm{e}} + 2i\Delta} \tag{9}$$

with detuning from the resonance frequency

$$\Delta = \omega - \omega_0. \tag{10}$$

For the RF resonator, we get fully equivalently

$$S_{11}^{\mathrm{RF}} = 1 - \frac{2\Gamma_{\mathrm{e}}}{\Gamma_{\mathrm{i}} + \Gamma_{\mathrm{e}} + 2i\Delta_0} \tag{11}$$

with $\Delta_0 = \Omega - \Omega_0$.

Real response function and fitting routine

When analyzing the measured cavity response, we consider a frequency-dependent complex-valued reflection background with amplitude and phase modulations originating from a variety of microwave components in our input and output lines and possible interfering signal paths. Under this assumption, we model the modified cavity response with

$$S_{11} = (\alpha_0 + \alpha_1\omega)\left(1 - \frac{2\kappa_{\mathrm{e}}e^{i\theta}}{\kappa_{\mathrm{i}} + \kappa_{\mathrm{e}} + 2i\Delta}\right)e^{i(\beta_1\omega + \beta_0)} \tag{12}$$

where we consider a frequency dependent complex background

$$S_{11} = (\alpha_0 + \alpha_1\omega)e^{i(\beta_1\omega + \beta_0)} \tag{13}$$

and an additional rotation of the resonance circle with the phase factor $e^{i\theta}$.
The first step in the fitting routine removes the cavity resonance part from the data curve and fits the remaining background with Eq. (13). After removing the background contribution from the full dataset by complex division, the resonator response is fitted using the ideal response function. In the final step, the full function is re-fitted to the bare data using as starting parameters the individually obtained fit numbers from the first two steps. From this final fit, we extract the final background fit parameters and remove the background of the full dataset by complex division. Also, we correct for the additional rotation factor $e^{i\theta}$. As result we obtain clean resonance curves as shown in Fig. 1**d** of the main paper.

Complex fits and mode parameters at the operation point

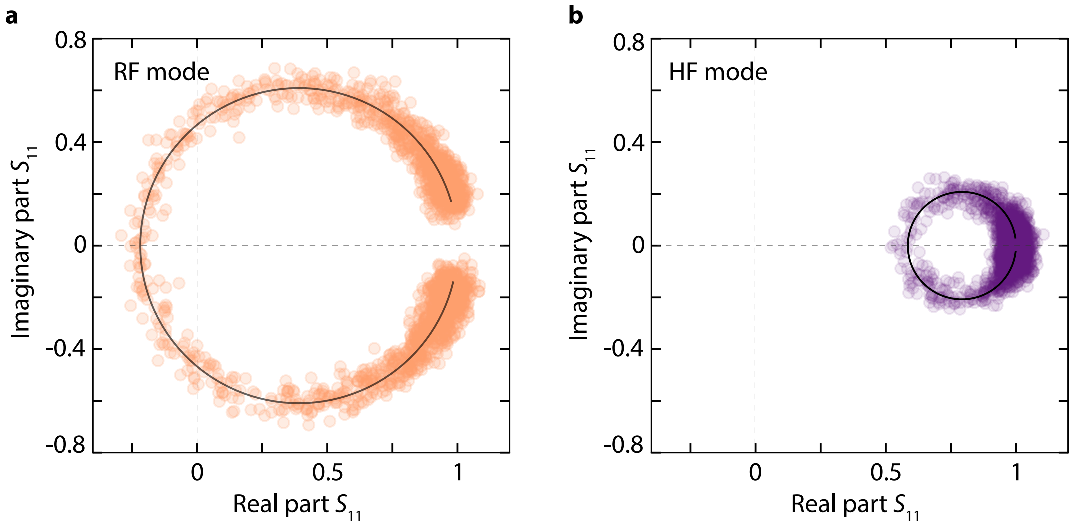

Supplementary Figure 8. **RF and HF complex resonance fits.** In **a** the resonance of the radio-frequency mode is shown, in **b** the resonance of the high-frequency mode. Circles are data, line is a fit. Extracted fit parameters are given in the text.

In Supplementary Fig. 8 we show the complex reflection signal obtained from the measurements for the RF mode in **a** and the HF mode in **b**. The background has been removed by complex division and the corresponding fit curves are added as lines. From the fits, we extract for the radio-frequency mode data shown in **a** the resonance frequency $\Omega_0 = 2\pi \cdot 452.5\,\mathrm{MHz}$, and the linewidths $\Gamma_\mathrm{i} = 2\pi \cdot 10\,\mathrm{kHz}$ and $\Gamma_\mathrm{e} = 2\pi \cdot 16\,\mathrm{kHz}$. The fit parameters of the high-frequency mode data shown in **b** are $\omega_0 = 2\pi \cdot 7.207\,\mathrm{GHz}$, $\kappa_\mathrm{i} = 2\pi \cdot 310\,\mathrm{kHz}$ and $\kappa_\mathrm{e} = 2\pi \cdot 80\,\mathrm{kHz}$ The absolute values of these two resonance are shown in main paper Fig. 1**d**.

SUPPLEMENTARY NOTE 5: ZERO-POINT FLUCTUATIONS Φ_zpf AND COUPLING RATE g_0

The zero-point current fluctuations of the radio-frequency mode at the operation point are given by

$$I_\mathrm{zpf} = \sqrt{\frac{\hbar\Omega_0}{2L_\mathrm{RF}}} \approx 28\,\mathrm{nA}, \tag{14}$$

where $\Omega_0 = 2\pi \cdot 452.5\,\mathrm{MHz}$ and $L_\mathrm{RF} = 188\,\mathrm{pH}$.
In presence of this zero-point current, which flows asymmetrically through the loop wire arm and the junction arm of the SQUID, the total flux in the SQUID is given by

$$\Phi = \Phi_\mathrm{b} + L_\mathrm{loop} J - \alpha L_\mathrm{loop}\frac{I_\mathrm{zpf}}{2} \tag{15}$$

where $\alpha = \frac{2L_\mathrm{a} - L_\mathrm{l}}{L_\mathrm{loop}} \approx 0.3$ describes the inductance asymmetry of the SQUID from the perspective of the RF currents. With the current-phase relation of the JJ, this can also be written as

$$\Phi = \Phi_\mathrm{b} + L_\mathrm{loop}(1-\alpha)\frac{I_\mathrm{zpf}}{2} - L_\mathrm{loop} I_\mathrm{c} \sin\left(\pi\frac{\Phi}{\Phi_0}\right). \tag{16}$$

Therefore, the zero-point fluctuation current is formally equivalent to a fluctuating external flux with

$$\begin{aligned}\Phi_\mathrm{zpf} &= L_\mathrm{loop}(1-\alpha)\frac{I_\mathrm{zpf}}{2} && (17)\\ &= L_\mathrm{l} I_\mathrm{zpf} && (18)\\ &\approx 635\,\mu\Phi_0. && (19)\end{aligned}$$

Using the derivative $\frac{\partial\omega_0}{\partial\Phi_\mathrm{b}}$ of the flux-dependence fit curve $\omega_0(\Phi_\mathrm{b})$, we can therefore calculate the single-photon coupling strength

$$g_0 = \frac{\partial\omega_0}{\partial\Phi_\mathrm{b}}\Phi_\mathrm{zpf}, \tag{20}$$

the result is shown in Supplementary Fig. 9.
At the bias point used in the reported experiments, we get $g_0 \approx 2\pi \cdot 160\,\mathrm{kHz}$. Around $\Phi_\mathrm{b}/\Phi_0 \sim 0.7$ though we get $g_0 \sim \kappa$, cf. also Supplementary Fig. 7. Also, for the regime $\Phi_\mathrm{b}/\Phi_0 > 0.5$, we get $g_0 \gtrsim \frac{\kappa+\Gamma_0}{4}$, i.e, the regime where a single red-sideband photon will induce well-resolved, parametric normal-mode splitting [26, 57].
From a comparison of the power calibration presented in Supplementary Note 2 with the intracavity photons required to obtain the observed total coupling rates $g = \sqrt{n_\mathrm{c}}g_0$ in Figs. 2-4 of the main paper we find only a very small disagreement in input line attenuation/intracavity pump photon number of $\sim 0.3\,\mathrm{dB}$. If we assume this disagreement to originate from uncertainties in the calculation of g_0 and Φ_zpf, respectively, we find the magnitude of the relative error in Φ_zpf to be 0.04.

SUPPLEMENTARY NOTE 6: PHOTON-PRESSURE SIDEBAND COOLING

Equations of motion

We model the (approximately red-sideband) driven system with the linearized equations of motion for photon-pressure interacting harmonic oscillators [1, 20]

$$\delta\dot{\hat{a}} = \left(-i\Delta - \frac{\kappa}{2}\right)\delta\hat{a} + ig(\hat{b} + \hat{b}^\dagger) + \sqrt{\kappa_\mathrm{i}}\hat{S}_\mathrm{i}^\mathrm{HF} + i\sqrt{\kappa_\mathrm{e}}\hat{S}_\mathrm{e}^\mathrm{HF} \tag{21}$$

$$\dot{\hat{b}} = \left(i\Omega_0 - \frac{\Gamma_0}{2}\right)\hat{b} + ig(\delta\hat{a} + \delta\hat{a}^\dagger) + \sqrt{\Gamma_\mathrm{i}}\hat{S}_\mathrm{i}^\mathrm{RF} + i\sqrt{\Gamma_\mathrm{e}}\hat{S}_\mathrm{e}^\mathrm{RF}. \tag{22}$$

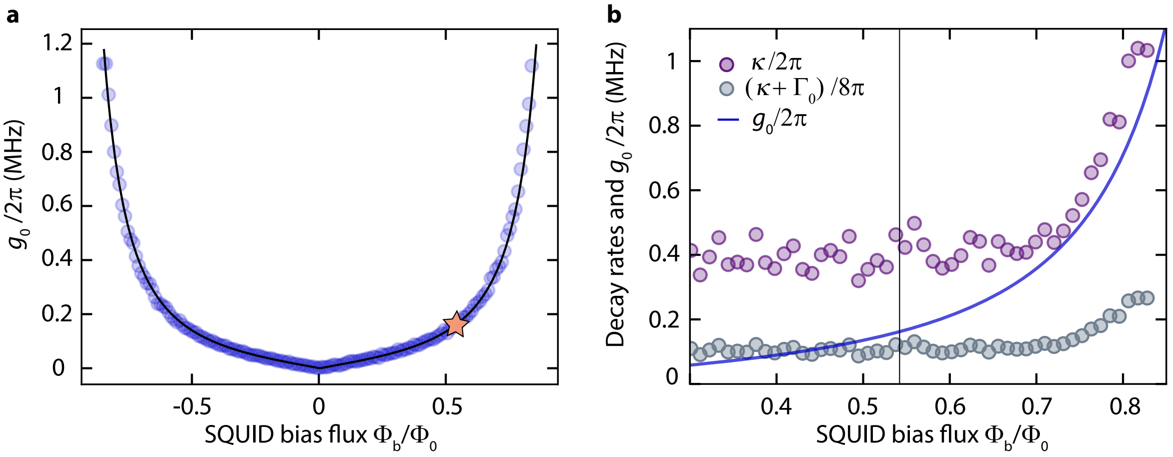

Supplementary Figure 9. **Single photon-coupling rate g_0 and decay rates vs SQUID bias flux.** **a** The points are calculated from the experimentally determined flux arch, the line is based on the arch fit, cf. main paper Fig. 1. For both, we calculate the derivative and multiply with the theoretical value for $\Phi_{\mathrm{zpf}} = 635\,\mu\Phi_0$. **b** shows the theoretically obtained g_0, the extracted cavity decay rate κ and $(\kappa+\Gamma_0)/4$ versus SQUID bias flux. The last quantity describes the g_0 limit for where a single sideband photon will induce parametric normal mode splitting. The operation point for the experiments reported here $\Phi/\Phi_0 = 0.54$ is marked by a star in **a** and as vertical gray line in **b**.

Here, $\delta\hat{a}$ and $\delta\hat{a}^\dagger$ describe the annihilation and creation operator for HF cavity field fluctuations, respectively, $\Delta = \omega_\mathrm{p} - \omega_0$, $g = \sqrt{n_\mathrm{c}}g_0$, and $\hat{S}_\mathrm{i}^\mathrm{HF}$ and $\hat{S}_\mathrm{e}^\mathrm{HF}$ corresponds to internal and external HF noise input fields. The RF mode annihilation and creation operators are given by $\hat{b}$ and $\hat{b}^\dagger$ and the internal and external RF noise input fields are taken into account by $\hat{S}_\mathrm{i}^\mathrm{RF}$ and $\hat{S}_\mathrm{e}^\mathrm{RF}$. The input noise operators $\hat{S}$ follow $\left\langle \hat{S}^\dagger\hat{S}\right\rangle = n$ and $\left\langle \hat{S}\hat{S}^\dagger\right\rangle = n+1$.

These equations can be solved by Fourier transform and the solutions read in frequency space

$$\delta\hat{a}(\Omega) = ig\chi_\mathrm{c}\left[\hat{b}(\Omega) + \hat{b}^\dagger(-\Omega)\right] + \chi_\mathrm{c}\left[\sqrt{\kappa_\mathrm{i}}\hat{S}_\mathrm{i}^\mathrm{HF}(\Omega) + i\sqrt{\kappa_\mathrm{e}}\hat{S}_\mathrm{i}^\mathrm{HF}(\Omega)\right] \tag{23}$$

$$\hat{b}(\Omega) = ig\chi_0\left[\delta\hat{a}(\Omega) + \delta\hat{a}^\dagger(-\Omega)\right] + \chi_0\left[\sqrt{\Gamma_\mathrm{i}}\hat{S}_\mathrm{i}^\mathrm{RF}(\Omega) + i\sqrt{\Gamma_\mathrm{e}}\hat{S}_\mathrm{e}^\mathrm{RF}(\Omega)\right] \tag{24}$$

with the susceptibilities

$$\chi_\mathrm{c} = \frac{1}{\frac{\kappa}{2} + i(\Delta+\Omega)} \tag{25}$$

$$\chi_0 = \frac{1}{\frac{\Gamma_0}{2} + i\Delta_0}, \tag{26}$$

Ω being the frequency relative to the pump tone and $\Delta_0 = \Omega - \Omega_0$.

Simplified equations of motion in the sideband-resolved regime with red-sideband pumping

Under red-sideband pumping $\Delta \approx -\Omega_0$ and in the sideband-resolved regime $\Omega_0 \gg \kappa$ the equations of motion can be simplified as

$$\delta\hat{a}(\Omega) = ig\chi_\mathrm{c}\hat{b}(\Omega) + \chi_\mathrm{c}\left[\sqrt{\kappa_\mathrm{i}}\hat{S}_\mathrm{i}^\mathrm{HF}(\Omega) + i\sqrt{\kappa_\mathrm{e}}\hat{S}_\mathrm{i}^\mathrm{HF}(\Omega)\right] \tag{27}$$

$$\hat{b}(\Omega) = ig\chi_0\delta\hat{a}(\Omega) + \chi_0\left[\sqrt{\Gamma_\mathrm{i}}\hat{S}_\mathrm{i}^\mathrm{RF}(\Omega) + i\sqrt{\Gamma_\mathrm{e}}\hat{S}_\mathrm{e}^\mathrm{RF}(\Omega)\right] \tag{28}$$

Solution for the RF mode response function

To calculate the response to a radio-frequency probe tone, we replace the noise input by a probe tone input $\hat{S}_0$ and get

$$\delta\hat{a}(\Omega) = ig\chi_c\hat{b}(\Omega) \tag{29}$$

$$\hat{b}(\Omega) = ig\chi_0\delta\hat{a}(\Omega) + i\chi_0\sqrt{\Gamma_e}\hat{S}_0(\Omega) \tag{30}$$

For the response function and the input-output relations [1] we find from this the result

$$S_{11}^{\mathrm{RF}} = 1 - \Gamma_e\frac{\chi_0}{1+g^2\chi_c\chi_0}. \tag{31}$$

The resonance condition $\left(\chi_0^{\mathrm{eff}}\right)^{-1} = 0$ for effective RF susceptibility

$$\chi_0^{\mathrm{eff}} = \frac{\chi_0}{1+g^2\chi_c\chi_0} \tag{32}$$

delivers the complex solutions

$$\tilde{\Omega}_\pm = \Omega_0 - \frac{\delta}{2} + i\frac{\kappa+\Gamma_0}{4} \pm \sqrt{g^2 - \left(\frac{\kappa-\Gamma_0+2i\delta}{4}\right)^2} \tag{33}$$

where δ is the pump detuning from the red sideband defined by $\Delta = -\Omega_0 + \delta$.

Solution for the HF mode response function

In full analogy to the RF mode, we get as probe tone response function for the HF mode

$$S_{11}^{\mathrm{HF}} = 1 - \kappa_e\frac{\chi_c}{1+g^2\chi_c\chi_0}. \tag{34}$$

with the complex solutions

$$\tilde{\omega}_\pm = \omega_0 + \frac{\delta}{2} + i\frac{\kappa+\Gamma_0}{4} \pm \sqrt{g^2 - \left(\frac{\kappa-\Gamma_0+2i\delta}{4}\right)^2} \tag{35}$$

Solution for the HF mode thermal noise power spectral density

We solve Eqs. (27), (28) with noise input now and get

$$\delta\hat{a} = \frac{ig\chi_c\chi_0\left[\sqrt{\Gamma_i}\hat{S}_i^{\mathrm{RF}} + i\sqrt{\Gamma_e}\hat{S}_e^{\mathrm{RF}}\right] + \chi_c\left[\sqrt{\kappa_i}\hat{S}_i^{\mathrm{HF}} + i\sqrt{\kappa_e}\hat{S}_e^{\mathrm{HF}}\right]}{1+g^2\chi_c\chi_0}. \tag{36}$$

which leads to the symmetrized output field power spectral density in units of photons [55]

$$\frac{S(\omega)}{\hbar\omega} = \frac{1}{2} + n_e^{\mathrm{HF}} + \kappa_e\kappa_i\frac{|\chi_c(\omega)|^2}{|1+g^2\chi_0(\Omega)\chi_c(\omega)|^2}\left(n_i^{\mathrm{HF}} - n_e^{\mathrm{HF}}\right) + \kappa_e\Gamma_0\frac{g^2|\chi_c(\omega)|^2|\chi_0(\Omega)|^2}{|1+g^2\chi_0(\Omega)\chi_c(\omega)|^2}\left(n_{\mathrm{th}}^{\mathrm{RF}} - n_e^{\mathrm{HF}}\right) \tag{37}$$

where the effective thermal photon occupation of the RF mode is given by the weighted sum

$$n_{\mathrm{th}}^{\mathrm{RF}} = \frac{\Gamma_i}{\Gamma_0}n_i^{\mathrm{RF}} + \frac{\Gamma_e}{\Gamma_0}n_e^{\mathrm{RF}}. \tag{38}$$

Added noise

The effective number of added noise photons by the amplifier chain is given by [20]

$$n'_{\mathrm{add}} = \frac{n_{\mathrm{add}}}{\eta} + \left(\frac{1-\eta}{\eta}\right)\frac{1}{2} \tag{39}$$

where n_{add} is the actual number of photons added by the HEMT amplifier noise in our case, and $\eta \sim 0.5$ accounts for losses of the cavity output field on its way to the HEMT. We will estimate the number of added noise photons based on a temperature sweep calibration presented below. As a rough first estimate, we can use the datasheet noise temperature of the amplifier of $\sim 2\,\mathrm{K}$ to find $n_{\mathrm{add}} \approx 5.3$ and $n'_{\mathrm{add}} \approx 11.1$.

The total power spectral density

For the power spectral density in units of photons at frequency $\omega = \omega_{\mathrm{p}} + \Omega$ of the SQUID cavity with a drive around the red sideband, we get for $n'_{\mathrm{add}}, n_{\mathrm{th}}^{\mathrm{RF}} \gg n_{\mathrm{e}}^{\mathrm{HF}}, n_{\mathrm{i}}^{\mathrm{HF}} \ll 1/2$ (where the latter corresponds to the reasonable assumption $T_{\mathrm{e}}^{\mathrm{HF}}, T_{\mathrm{i}}^{\mathrm{HF}} \lesssim 100\,\mathrm{mK}$)

$$\frac{S(\omega)}{\hbar\omega} = \frac{1}{2} + n'_{\mathrm{add}} + \kappa_{\mathrm{e}}\Gamma_0 \frac{g^2|\chi_{\mathrm{c}}(\omega)|^2|\chi_0(\Omega)|^2}{|1+g^2\chi_0(\Omega)\chi_{\mathrm{c}}(\omega)|^2} n_{\mathrm{th}}^{\mathrm{RF}} \tag{40}$$

This can be also written as

$$\frac{S(\omega)}{\hbar\omega} = \frac{1}{2} + n'_{\mathrm{add}} + \frac{16\kappa_{\mathrm{e}} g^2 \Gamma_0 n_{\mathrm{th}}^{\mathrm{RF}}}{\left|4g^2 + [\kappa + 2i(\delta+\Delta_0)]\,[\Gamma_0 + 2i\Delta_0]\right|^2} \tag{41}$$

where $\Delta_0 = \Omega - \Omega_0$ takes into account the detuning from the RF resonance frequency and $\delta = \omega_{\mathrm{p}} - (\omega_0 - \Omega_0)$ takes into account the detuning of the pump from the red sideband of the cavity. By fitting the measured power spectral density with Eq. (41), as shown as fit curves in Fig. 3**c** of the main paper, we obtain for each curve the thermal photon number occupancy of the RF mode as detailed below.

Cooled RF photons

For the RF mode we get from the equations of motion

$$\hat{b} = \frac{ig\chi_{\mathrm{c}}\chi_0\left[\sqrt{\kappa_{\mathrm{i}}}\hat{S}_{\mathrm{i}}^{\mathrm{HF}} + i\sqrt{\kappa_{\mathrm{e}}}\hat{S}_{\mathrm{e}}^{\mathrm{HF}}\right] + \chi_0\left[\sqrt{\Gamma_{\mathrm{i}}}\hat{S}_{\mathrm{i}}^{\mathrm{RF}} + i\sqrt{\Gamma_{\mathrm{e}}}\hat{S}_{\mathrm{e}}^{\mathrm{RF}}\right]}{1+g^2\chi_{\mathrm{c}}\chi_0}. \tag{42}$$

We can use this to calculate the RF photon population with a sideband drive exactly on the red sideband and get

$$n_{\mathrm{cool}}^{\mathrm{RF}} = \frac{\Gamma_0}{\kappa+\Gamma_0}\frac{4g^2+\kappa(\kappa+\Gamma_0)}{4g^2+\kappa\Gamma_0} n_{\mathrm{th}}^{\mathrm{RF}} + \frac{\kappa}{\kappa+\Gamma_0}\frac{4g^2}{4g^2+\kappa\Gamma_0} n_{\mathrm{th}}^{\mathrm{HF}}. \tag{43}$$

Compared to the usually quoted result [1, 20, 56], we find some corrections in the cooled RF occupation, in particular the appearance of the factor $\kappa + \Gamma_0$ instead of κ. These corrections are negligble for $\kappa \gg \Gamma_0$, which in our case, however is not strictly true anymore.
For non-vanishing detuning we get

$$n_{\mathrm{cool}}^{\mathrm{RF}} = \frac{\Gamma_0}{\kappa+\Gamma_0}\frac{4g^2+\kappa(\kappa+\Gamma_0)\left[1+\frac{4\delta^2}{(\kappa+\Gamma_0)^2}\right]}{4g^2+\kappa\Gamma_0\left[1+\frac{4\delta^2}{(\kappa+\Gamma_0)^2}\right]} n_{\mathrm{th}}^{\mathrm{RF}} + \frac{\kappa}{\kappa+\Gamma_0}\frac{4g^2}{4g^2+\kappa\Gamma_0\left[1+\frac{4\delta^2}{(\kappa+\Gamma_0)^2}\right]} n_{\mathrm{th}}^{\mathrm{HF}}. \tag{44}$$

SUPPLEMENTARY NOTE 7: TEMPERATURE CALIBRATION

To perform a calibration of the RF resonator thermal occupation, we vary the fridge temperature T_{f} in steps of $20\,\mathrm{mK}$ and take a series of measurements for each T_{f}. During this procedure, we keep the flux bias constant at $\Phi_{\mathrm{b}}/\Phi_0 = 0.54$.

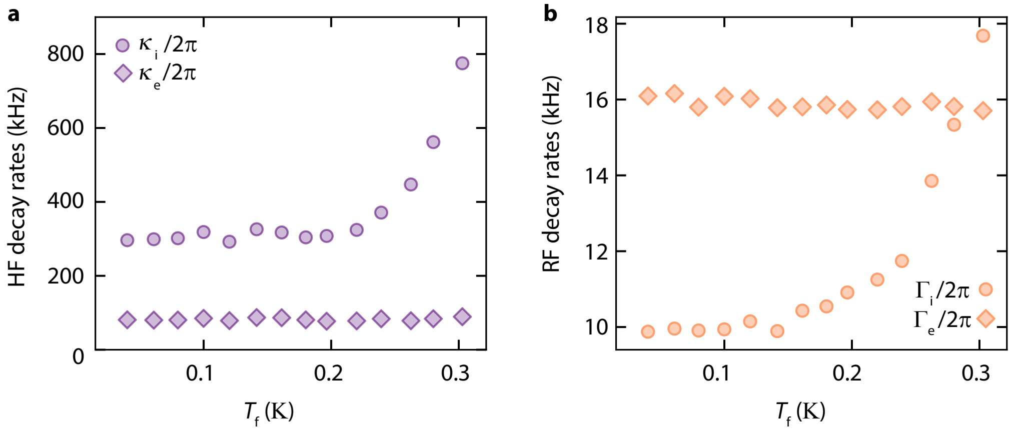

Supplementary Figure 10. **Linewidths vs temperature.** Decay rates of the microwave mode κ, κ_e in panel **a** and of the radio-frequency mode Γ_i and Γ_e in panel **b** depending on fridge temperature T_f.

First, we take bare response measurements of the two modes S_{11}^{RF} and S_{11}^{HF}. From the response curves, we extract the resonance frequencies ω_0 and Ω_0 as well as the linewidths κ, κ_e and Γ_i, Γ_e by fitting the responses using Eq. (9). The linewidths are shown in Supplementary Fig. 10.
Afterwards, we set a pump tone of constant power to the red sideband of the HF cavity and detect both, the photon-pressure induced transparency (PPIT) in a reflection measurement of the HF cavity and the upconverted RF thermal noise power spectrum. We adjust the pump power for each temperature to keep the cooperativity $\mathcal{C} \leq 1$ in order to avoid being too close to the normal-mode splitting regime but still obtain a good signal. From the PPIT data, we determine the actual cooperativity. Using the resonance frequencies and the linewidths, we determine the thermal photon occupation number in the RF mode relative to the added noise photons from the detected thermal noise curve for each temperature as described in Supplementary Note 6.
We repeat the output noise detection for two distinct experimental conditions which correspond to two different RF mode temperatures. The feedline of the RF circuit is connected to a cryogenic radio-frequency amplifier and as we do not have a radio-frequency isolator or circulator, the noise emitted by this amplifier will reach the RF input with only small attenuation. Hence, we can increase or decrease the RF input noise by switching this amplifier on or off, respectively, cf. Supplementary Fig 11**b**.
As first step in the data analysis and temperature calibration, we use the "amplifier off" dataset, i.e., the "cold" RF mode to estimate the number of added photons $n'_{\rm add}$. We experimentally observe that the effective RF mode occupation increases almost linearly for the larger fridge temperatures and saturates at a constant value for low fridge temperatures, cf. Supplementary Fig. 11**c** or main paper Fig. 3**b**. This behaviour indicates that for large fridge temperatures the RF bath follows the base temperature, but that for low temperatures the RF bath temperature is not completely equilibrating to the fridge. To capture this observation phenomenologically, we assume the effective bath temperature to be given by $T_{\rm RF} = \sqrt{T_{\rm f}^2 + T_{\rm r}^2}$ with the fridge temperature $T_{\rm f}$ and the residual mode temperature $T_{\rm r}$. The resulting RF mode occupation is then given by

$$n^{\rm RF} = \left(e^{\frac{\hbar\omega_0}{k_B T_{\rm RF}}} - 1\right)^{-1}. \qquad (45)$$

This function will lead to a curve very similar to the observations, i.e., to a linear increase of $n^{\rm RF}$ for large base temperatures and to a gradual saturation at a residual occupation at low temperatures at $T_{\rm RF} \approx T_{\rm r} \gg T_{\rm f}$. We use the normalized occupation data extracted from fits to the experimental power spectral densities $\tilde{n} = n_{\rm th}^{\rm RF}(T_{\rm f})/\left(n'_{\rm add} + \frac{1}{2}\right)$ using Eq. (41) now and fit these extracted and normalized data points with the function $\alpha n^{\rm RF}(T_{\rm f})$ with $\alpha = (n'_{\rm add} + 1/2)^{-1}$ using $T_{\rm r}$ and α as fit parameters. We obtain $T_{\rm r} \approx 141\,$mK and $n'_{\rm add} = \frac{1}{\alpha} - \frac{1}{2} \approx 10.6$. However, our results depend slightly on the chosen function for the effective temperature. We can get a similar behaviour for any function $T_{\rm RF} = \left(T_{\rm f}^k + T_{\rm r}^k\right)^{\frac{1}{k}}$ with k being approximately in the range $1.5 \leq k \leq 4$ and which leads to $n'_{\rm add}$ values in the range $9 \leq n'_{\rm add} \leq 12$. To take this spread into account, which arises from using only a phenomenological model function, we will use an average value of $n'_{\rm add} \approx 11 \pm 2$. This value for $n'_{\rm add}$ is consistent with an HF HEMT noise temperature of $\sim 2\,$K (datasheet) and $2.7\,$dB attenuation between sample and amplifier.
The results we obtain with $n'_{\rm add} = 11$ for the RF mode occupation in both RF amplifier configurations are shown in Supplementary Fig. 11**c**. For the RF amplifier switched off, we obtain a residual occupation of 7 ± 1 photons at the

fridge base temperature, which stays nearly constant until the fridge reaches about 150 mK. Note, that this residual value of ~ 7 photons is considerably larger than what would be theoretically expected from the Bose distribution for a complete thermalization to the base temperature, which would be $n_{\mathrm{Bose}}^{\mathrm{RF}}(20\,\mathrm{mK}) \approx 0.5$, cf. also black line in Supplementary Fig. 11**c**. We will discuss possible reasons for this deviation below. From around $100-150\,\mathrm{mK}$ upwards, the RF occupation starts to increase and approaches the Bose occupation shown as black line. For the configuration with the amplifier switched on, the residual occupation is about a factor of three larger due to the increased noise coming along the input/output line from the amplifier and coupling into the circuit. Still, a slight increase of the total occupation with increasing fridge temperature is visible, which can be attributed to an increase of the internal RF mode bath temperature.

In both amplifier states, the residual occupation of the RF mode is considerably larger than what is expected from the Bose factor and for full thermalization. The observation that the RF amplifier state (on vs off) has such a large impact, however, lets us believe that the main contribution to the deviation from the Bose occupation is the noise propagating along the RF feedline from the unisolated amplifier to the sample. The amplifier is mounted in between the 800 mK plate and the 3 K plate, and except for a directional coupler, a 1 dB attenuator and cable losses is not isolated further from the device, cf. also Supplementary Fig. 3. Although for a detailed and concise analysis of the origin and the mechanism behind this residual occupation, a precise knowledge of the individual bath temperatures and their dependence on the fridge temperature would be necessary, we will try to shed some light onto the internal and external contribution to the RF mode occupation with an extension of the phenomenological model used already above. To model the thermal occupation taking into account contributions from internal and external baths, we use

$$n_{\mathrm{th}}^{\mathrm{RF}} = \frac{\Gamma_{\mathrm{i}}}{\Gamma_0} n_{\mathrm{i}}^{\mathrm{RF}} + \frac{\Gamma_{\mathrm{e}}}{\Gamma_0} n_{\mathrm{e}}^{\mathrm{RF}} \tag{46}$$

where the internal and external occupations are given by

$$n_{\mathrm{i/e}}^{\mathrm{RF}} = \left(e^{\frac{\hbar\omega_0}{k_{\mathrm{B}} T_{\mathrm{i/e}}}} - 1 \right)^{-1}. \tag{47}$$

For the effective internal and external temperatures T_{i} and T_{e}, respectively, we take into account possible devations from the fridge temperature by a residual temperature $T_{\mathrm{r,i/e}}$, which e.g. considers the RF amplifier noise arriving at the sample RF input. To phenomenologiclly model a gradual adjustment of the effective bath temperatures to the fridge temperature, we use $T_{\mathrm{i/e}} = \sqrt{T_{\mathrm{f}}^2 + T_{\mathrm{r,i/e}}^2}$. In addition, we fit the temperature dependence of Γ_{i} and take it into account in the calculation of $n_{\mathrm{th}}^{\mathrm{RF}}$. For the case of RF amplifier off, we observe an increased RF mode linewidth $\Gamma_0 \approx 2\pi \cdot 40\,\mathrm{kHz}$, which we attribute to two-level systems being saturated with the amplifier on. With all these factors considered, we obtain the lines shown in Fig. 11**c** from a simultaneous fit of the ON and OFF datasets, giving good qualitative agreement with the data. As fit parameters, we used $T_{\mathrm{r,i}}, T_{\mathrm{r,e}}^{\mathrm{on}}$ and $T_{\mathrm{r,e}}^{\mathrm{off}}$. Hence, the parameter set for both curves is identical, except for the residual temperature of the external bath $T_{\mathrm{r,e}}$, which is modified by the amplifier power state, and for Γ_0, which differs between the amplifier ON and OFF states. The extracted temperatures are $T_{\mathrm{r,i}} = 31\,\mathrm{mK}$, $T_{\mathrm{r,e}}^{\mathrm{on}} = 743\,\mathrm{mK}$ and $T_{\mathrm{r,e}}^{\mathrm{off}} = 285\,\mathrm{mK}$. Furthermore, the internal and external linewidths at base temperature were fixed to $\Gamma_{\mathrm{e}} = 2\pi \cdot 16\,\mathrm{kHz}$, $\Gamma_{\mathrm{i}}^{\mathrm{on}} = 2\pi \cdot 10\,\mathrm{kHz}$, $\Gamma_{\mathrm{i}}^{\mathrm{off}} = 2\pi \cdot 24\,\mathrm{kHz}$. Within this model, we find our intuitive explanation confirmed, i.e., that the noise propagating from the hot RF amplifier to the device is the main origin for the large residual occupation at base temperature. We also note, however, that the results of this fitting depend significantly on the exact model used for the effective bath temperatures and that more refined experiments would be required to fully and reliably determine the mechanisms and noise processes of the RF mode.

SUPPLEMENTARY NOTE 8: COOLING THE RF MODE WITH INCREASED THERMAL OCCUPANCY

We repeat the cooling experiment discussed in the main paper Fig. 3 also for the RF amplifier switched on, leaving the RF mode occupied with about 20.5 thermal photons at fridge base temperature. The resulting spectra of the sideband-cooling in this state are shown in Supplementary Fig. 11 and besides a larger amplitude due to the increased occupancy look nearly identical to the spectra shown in the main paper. The corresponding cooled RF photons are plotted in **e** together with the data for the amplifier switched off.

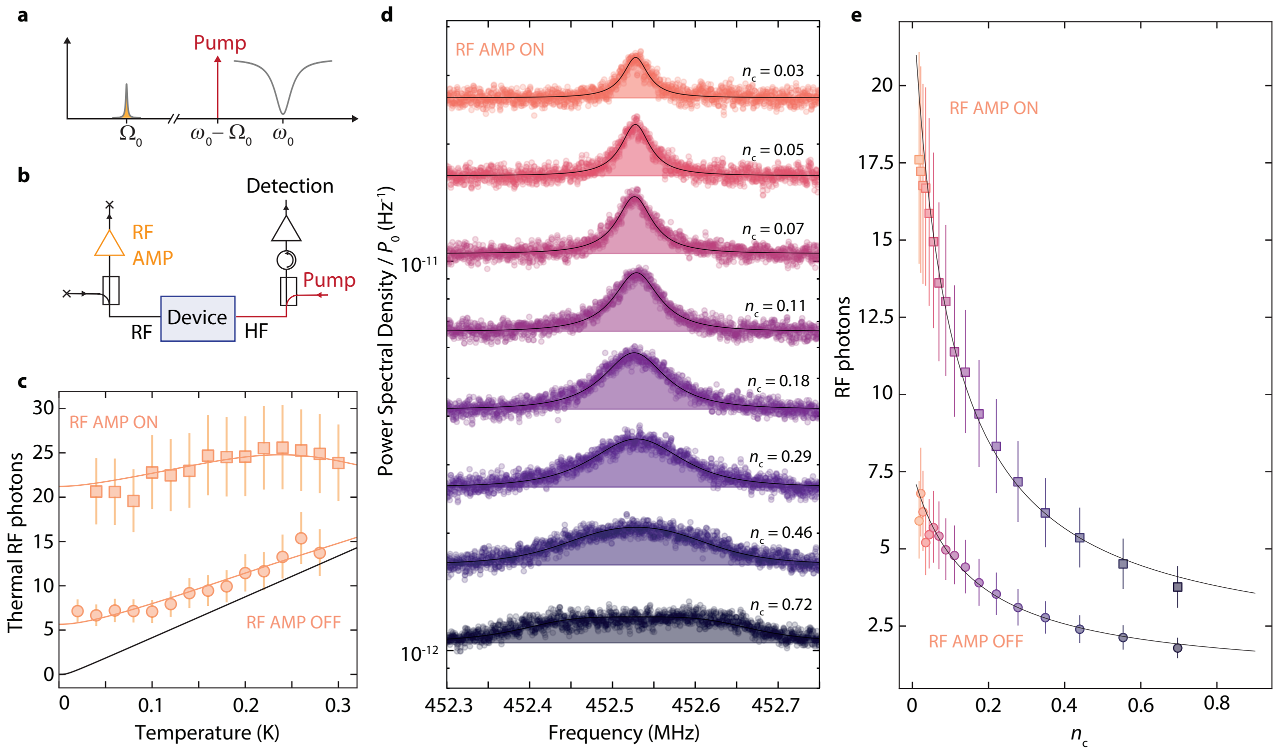

Supplementary Figure 11. **Photon-pressure sideband-cooling of a hot and a hotter RF resonator.** **a** For the observation of upconverted thermal noise of the RF resonator, a pump tone is set to the red sideband of the high-frequency mode $\omega_\mathrm{p} = \omega_0 - \Omega_0$ and the cavity output field around $\omega = \omega_0$ is detected with a signal analyzer. The RF input/output side of the device is connected to a cryogenic radio-frequency amplifier, which is used for the reflection characterization of the RF mode, cf. panel **b**. The state of this RF amplifier can be used to control the thermal occupation of the RF mode. When it is switched ON, its output noise increases the thermal occupation of the RF mode as shown in panel **c**, where the thermal photon number vs fridge temperature is plotted for both cases, RF amplifier switched ON and RF amplifier switched OFF. Symbols are data, black line is the Bose factor, orange lines are models for the thermal occupation and discussed in the text. From the thermal calibration, we determine the thermal occupation of the RF mode at fridge base temperature to be $n_\mathrm{RF}^\mathrm{on} \sim 20.5$ and $n_\mathrm{RF}^\mathrm{off} \sim 7$. **d** shows the measured high-frequency output power spectral density for increasing red-sideband pump power, normalized to the on-chip pump power P_0 for RF amplifier ON. Frequency axis is given with respect to the constant pump frequency. Circles are data, lines and shaded areas are fits. With increasing pump strength, i.e., increasing intracavity photon number n_c, the RF resonance gets broadened by photon-pressure damping, and its total thermal noise power gets reduced, which corresponds to cooling of the mode. In **e**, the thermal RF mode occupation is shown as symbols vs pump photon number n_c for both cases, RF amplifier ON (squares) and RF amplifier OFF (circles). Error bars for the amplifier ON (OFF) data correspond to uncertainties of ± 2 HF photons of added noise in the detection chain and ± 1 kHz (± 2 kHz) in bare RF linewidth $\Gamma_0^\mathrm{on} = 26$ kHz ($\Gamma_0^\mathrm{off} = 40$ kHz).

SUPPLEMENTARY NOTE 9: THEORY OF NORMAL-MODE THERMOMETRY

The high-frequency response function with normal mode susceptibilities

From the equations of motion, we obtained the response of the system around the HF mode as

$$S_{11}^\mathrm{HF} = 1 - \kappa_\mathrm{e} \frac{\chi_\mathrm{c}}{1 + g^2 \chi_\mathrm{c} \chi_0} \tag{48}$$

under the assumption of pumping around the red sideband and the sideband-resolved regime. The resonance condition $\left(\chi_\mathrm{c}^\mathrm{eff}\right)^{-1} = 0$ for the effective HF cavity susceptibility

$$\chi_\mathrm{c}^\mathrm{eff} = \frac{\chi_\mathrm{c}}{1 + g^2 \chi_\mathrm{c} \chi_0} \tag{49}$$

provided us with the complex solutions of the effective susceptibility

$$\tilde{\omega}_{\pm} = \omega_0 + \frac{\delta}{2} + i\frac{\kappa + \Gamma_0}{4} \pm \sqrt{g^2 - \left(\frac{\kappa - \Gamma_0 + 2i\delta}{4}\right)^2} \tag{50}$$

where δ is the pump detuning from the red HF cavity sideband.
Now, we define the normal mode susceptibilities

$$\chi_+ = \frac{1}{\frac{\kappa_+}{2} + i\Delta_+}, \quad \chi_- = \frac{1}{\frac{\kappa_-}{2} + i\Delta_-} \tag{51}$$

where $\Delta_{\pm} = \omega - \omega_{\pm}$ and

$$\omega_{\pm} = \mathrm{Re}[\tilde{\omega_{\pm}}], \quad \kappa_{\pm} = \mathrm{Im}[\tilde{\omega_{\pm}}] \tag{52}$$

are the real and imaginary parts, respectively, of the complex solutions.
With these, we can rewrite the HF response function as

$$S_{11}^{\mathrm{HF}} = 1 - \kappa_{\mathrm{e},+}\chi_+ - \kappa_{\mathrm{e},-}\chi_- \tag{53}$$

which is exact with the (complex and frequency-dependent) external linewidths

$$\kappa_{\mathrm{e},\pm} = \mp\frac{i\kappa_{\mathrm{e}}}{2\chi_0\sqrt{g^2 - \left(\frac{\kappa - \Gamma_0 + 2i\delta}{4}\right)^2}}. \tag{54}$$

For the regime of considerable coupling $g \gg \kappa/2, \Gamma_0/2$ and possibly large detunings $\Delta \lesssim g$, we approximate this by

$$\kappa_{\mathrm{e},\pm} \approx \frac{\kappa_{\mathrm{e}}}{2}\left(1 \pm \frac{\delta}{\sqrt{\delta^2 + 4g^2}}\right). \tag{55}$$

Normal-mode thermometry

Using the approximated normal-mode representation of the HF cavity in the strong-coupling regime, we get for the output field power spectral density in units of quanta

$$\frac{S_{\mathrm{nms}}}{\hbar\omega} = \frac{1}{2} + n'_{\mathrm{add}} + 4\frac{\kappa_{\mathrm{e},+}\kappa_{\mathrm{i},+}}{\kappa_+^2 + 4\Delta_+^2}\left(n_{\mathrm{i},+} - n_{\mathrm{e},+}\right) + 4\frac{\kappa_{\mathrm{e},-}\kappa_{\mathrm{i},-}}{\kappa_-^2 + 4\Delta_-^2}\left(n_{\mathrm{i},-} - n_{\mathrm{e},-}\right) \tag{56}$$

where $\kappa_{\mathrm{i},\pm} = \kappa_{\pm} - \kappa_{\mathrm{e},\pm}$ and $n_{\mathrm{e},+}, n_{\mathrm{e},-}$ and $n_{\mathrm{i},+}, n_{\mathrm{i},-}$ are the effective external and internal bath occupations of the normal modes, respectively. If the external baths are given again by the fridge temperature, we get $n_{\mathrm{e},\pm} \ll n_{\mathrm{i},\pm} \approx \frac{\kappa_{\pm}}{\kappa_{\mathrm{i},\pm}} n_{\pm}$. From the condition $S_{\mathrm{nms}} = S$, we can then follow

$$\kappa_{\mathrm{e},+}n_+ + \kappa_{\mathrm{e},-}n_- = \kappa_{\mathrm{e}} n_{\mathrm{cool}}^{\mathrm{HF}} \tag{57}$$

where $n_{\mathrm{cool}}^{\mathrm{HF}}$ is the thermal occupation (imbalance) of the HF mode while cooling the RF mode with a red sideband tone. As $n_{\mathrm{cool}}^{\mathrm{HF}} \gg n_{\mathrm{e}}^{\mathrm{HF}}$, the imbalance occupation corresponds in good approximation to the total occupation.
So this way we can calculate the effective HF cavity occupation which is given by

$$n_{\mathrm{cool}}^{\mathrm{HF}} = \frac{\kappa}{\kappa + \Gamma_0}\frac{4g^2 + \Gamma_0(\kappa + \Gamma_0)\left[1 + \frac{4\delta^2}{(\kappa+\Gamma_0)^2}\right]}{4g^2 + \kappa\Gamma_0\left[1 + \frac{4\delta^2}{(\kappa+\Gamma_0)^2}\right]} n_{\mathrm{th}}^{\mathrm{HF}} + \frac{\Gamma_0}{\kappa + \Gamma_0}\frac{4g^2}{4g^2 + \kappa\Gamma_0\left[1 + \frac{4\delta^2}{(\kappa+\Gamma_0)^2}\right]} n_{\mathrm{th}}^{\mathrm{RF}}. \tag{58}$$

when red-sideband driving.
The total number of noise photons in the hybridized mode regime is then given by

$$n_{\mathrm{tot}} = n_{\mathrm{cool}}^{\mathrm{HF}} + n_{\mathrm{cool}}^{\mathrm{RF}}. \tag{59}$$

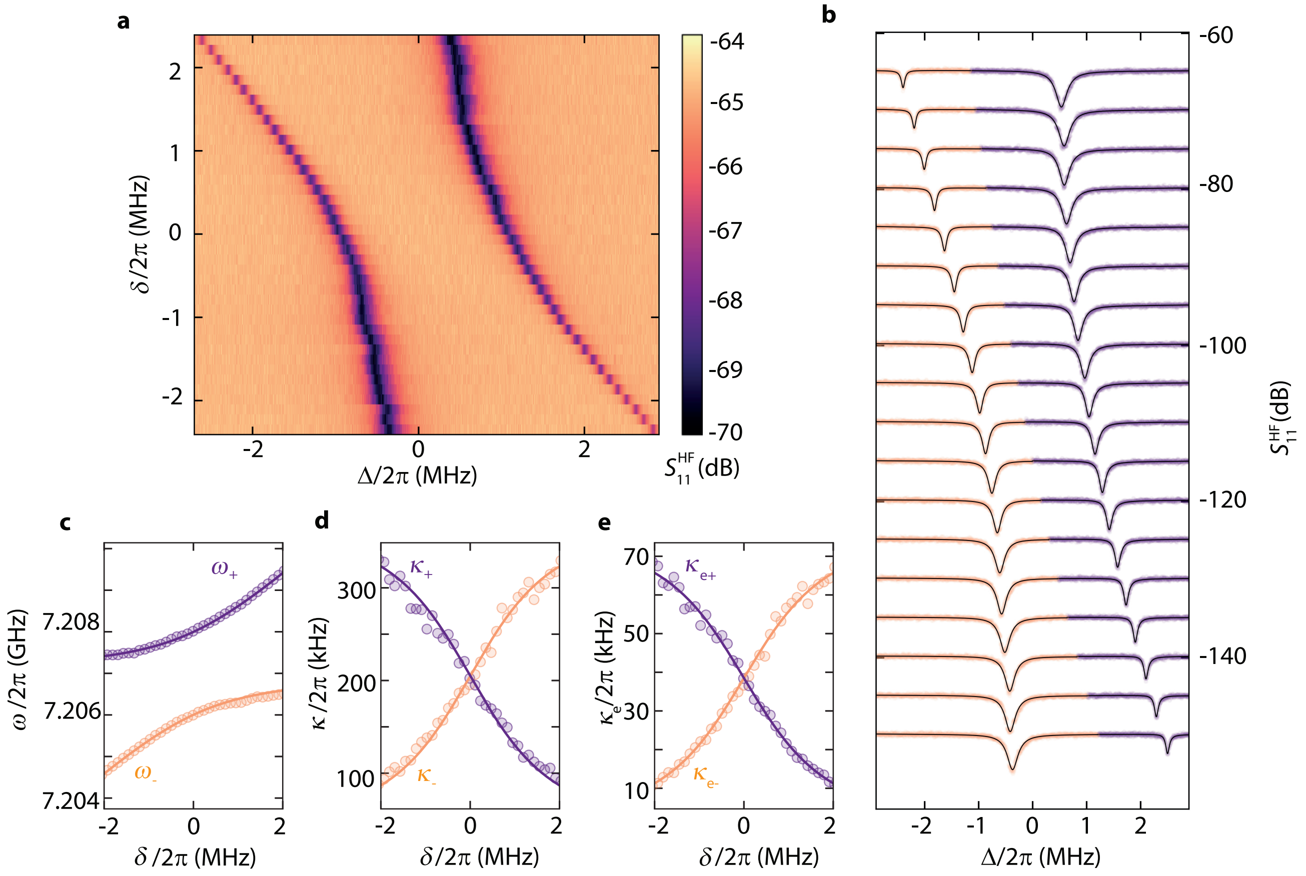

Supplementary Figure 12. **HF response in the strong-coupling regime and normal-mode fit parameters.** **a** Color-coded HF reflection response in the strong-coupling regime. The pump tone is swept through the HF mode red sideband with $\omega_\mathrm{p} = \omega_0 - \Omega_0 + \delta$. The response frequency is given relative to the bare HF cavity mode $\Delta = \omega - \omega_0$. **b** Linecuts of **a**, showing the individually fitted parts of the response as orange and purple. The top curve is plotted as measured (unshifted), subsequent curves are manually downshifted by -5 dB each for clarity. Shown is every second linescan of **a**. Fit curves are plotted as black lines. From the fit curves shown in **b**, we get the three relevant normal-mode parameters resonance frequency $\omega_\pm$, total decay rate $\kappa_\pm$ and external decay rate $\kappa_{\mathrm{e},\pm}$, plotted in **c**, **d**, and **e**, respectively.

SUPPLEMENTARY NOTE 10: DETERMINATION OF SYSTEM PARAMETERS FOR NORMAL-MODE THERMOMETRY

For the analysis of the normal-mode thermal spectra, it is essential to know the individual mode parameters, which we obtain from a characterization of the HF reflection response in the strong-coupling regime. Supplementary Fig. 12**a** shows S_{11}^HF for approximately $n_\mathrm{c} = 100$ pump intracavity photons. This data was measured simultaneously with the power spectral densities (PSD) shown in main paper Fig. 4, but during the measurement of the PSDs the network analyzer was switched off. Both datasets were acquired while iteratively sweeping a pump tone through the red sideband with $\omega_\mathrm{p} = \omega_0 - \Omega_0 + \delta$. As the pump tone approaches exactly the red sideband frequency, we observe photon-pressure induced hybridization between the modes with a resonant splitting of $g/\pi \sim 2.1\,\mathrm{MHz}$, about one order of magnitude larger than the normal-mode linewidths $(\kappa + \Gamma_0)/2 = 2\pi \cdot 200\,\mathrm{kHz}$ and a factor of three larger than the RF thermal decoherence rate $\Gamma_0 n_\mathrm{th}^\mathrm{RF} \approx 2\pi \cdot 300\,\mathrm{kHz}$.

For an analysis of the normal modes, we denote the lower-frequency mode with the superscript $-$ and the higher-frequency mode with $+$, i.e., the resonance frequencies are identified as ω_- and ω_+ and the total and external linewidths

as κ_-, κ_+ and $\kappa_{\mathrm{e},-}$, $\kappa_{\mathrm{e},+}$, respectively. The theoretical description of these parameters was given in Supplementary Note 9. To determine these parameters from the experimental data, each linescan of Supplementary Fig. 12**a** was split into two parts, each of them containing one of the two resonances. This splitting is shown in **b** by using two different colors, orange for the range of ω_- and purple for the range of ω_+. Both sub-responses were fitted individually using Eq. (9).

The returned fit parameters $\omega_\pm$, $\kappa_\pm$ and $\kappa_{\mathrm{e}\pm}$ are plotted in Supplementary Figs. 12**c**, **d**, and **e**, respectively. The theoretical dependences as derived in Supplementary Note 9 are plotted as lines and show excellent agreement with the data. When the two modes are completely hybridized ($\delta = 0$), we find for both a total decay rate $\kappa_+ = \kappa_- = 2\pi \cdot 200\,\mathrm{kHz}$, which is what we expect from the theoretical expression for this case $\kappa_\pm = (\kappa + \Gamma_0)/2$. Also the external decay rates, usually defined by the coupling capacitance to the feedline, are half the value of the bare HF mode $\kappa_{\mathrm{e},+} = \kappa_{\mathrm{e},-} = 2\pi \cdot 40\,\mathrm{kHz}$.
